# Effectiveness of Screening and Treatment Approaches for Schistosomiasis and Strongyloidiasis in Newly-Arrived Migrants from Endemic Countries in the EU/EEA: A Systematic Review

**DOI:** 10.3390/ijerph16010011

**Published:** 2018-12-20

**Authors:** Eric N. Agbata, Rachael L. Morton, Zeno Bisoffi, Emmanuel Bottieau, Christina Greenaway, Beverley-A. Biggs, Nadia Montero, Anh Tran, Nick Rowbotham, Ingrid Arevalo-Rodriguez, Daniel T. Myran, Teymur Noori, Pablo Alonso-Coello, Kevin Pottie, Ana Requena-Méndez

**Affiliations:** 1 Faculty of Health Science, University of Roehampton London, London SW15 5PU, UK; 2 Department of Paediatrics, Obstetrics, Gynaecology and Preventive Medicine, Universitat Autònoma de Barcelona, Bellaterra, 08193 Barcelona, Spain; 3 NHMRC Clinical Trials Centre, University of Sydney, Camperdown, NSW 2050, Australia; Rachael.morton@ctc.usyd.edu.au (R.L.M.); anh.tran@ctc.usyd.edu.au (A.T.); rowbothamn@gmail.com (N.R.); 4 Centre for Tropical Diseases (CTD), IRCCS Sacro Cuore Don Calabria Negrar, Negrar, 37024 Verona, Italy; zeno.bisoffi@sacrocuore.it; 5 Department of Diagnostics and Public Health, University of Verona, 37134 Verona, Italy; 6 Department of Clinical Sciences, Institute of Tropical Medicine, 155 Nationalestraat, 2000 Antwerp, Belgium; EBottieau@itg.be; 7 Division of Infectious Diseases and Clinical Epidemiology, Sir Mortimer B. Davis-Jewish General Hospital, McGill University, Montreal, QC H3A 0G4, Canada; ca.greenaway@mcgill.ca; 8 Department of Medicine at the Doherty Institute, University of Melbourne, Parkville, VIC 3010, Australia; babiggs@unimelb.edu.au; 9 Victorian Infectious Diseases Service, The Royal Melbourne Hospital RMH, Parkville VIC 3050, Australia; 10 Centro de Investigación en Salud Pública y Epidemiología Clínica (CISPEC), Facultad de Ciencias de la Salud Eugenio Espejo, Universidad Tecnológica Equinoccial, Quito 170509, Ecuador; nadiamonteromd@gmail.com (N.M.); inarev7@yahoo.com (I.A.-R.); 11 Clinical Biostatistics Unit, Hospital Universitario Ramon y Cajal (IRYCIS); CIBER Epidemiology and Public Health (CIBERESP), 28034 Madrid, Spain; 12 Bruyere Research Institute, University of Ottawa, Ottawa, ON K1N 6N5, Canada; daniel.myran@gmail.com; 13 European Centre for Disease Prevention and Control, Gustav III: s Boulevard 40, 169 73 Solna, Sweden; Teymur.Noori@ecdc.europa.eu; 14 Iberoamerican Cochrane Center, Biomedical Research Institute Sant Pau (IIB Sant Pau-CIBERESP), 08025 Barcelona, Spain; PAlonso@santpau.cat; 15 Centre for Global Health Institute of Population Health, University of Ottawa, Ottawa, ON K1N 6N5, Canada; kpottie@uottawa.ca; 16 ISGlobal, Barcelona Institute for Global Health (ISGlobal-CRESIB, Hospital Clínic-University of Barcelona), E-08036 Barcelona, Spain; ana.requena@isglobal.org

**Keywords:** migrant populations, schistosomiasis/schistosoma, strongyloidiasis/strongyloides, screening/diagnosis, treatment, public health, GRADE

## Abstract

We aimed to evaluate the evidence on screening and treatment for two parasitic infections—schistosomiasis and strongyloidiasis—among migrants from endemic countries arriving in the European Union and European Economic Area (EU/EEA). We conducted a systematic search of multiple databases to identify systematic reviews and meta-analyses published between 1 January 1993 and 30 May 2016 presenting evidence on diagnostic and treatment efficacy and cost-effectiveness. We conducted additional systematic search for individual studies published between 2010 and 2017. We assessed the methodological quality of reviews and studies using the AMSTAR, Newcastle–Ottawa Scale and QUADAS-II tools. Study synthesis and assessment of the certainty of the evidence was performed using GRADE (Grading of Recommendations Assessment, Development and Evaluation) approach. We included 28 systematic reviews and individual studies in this review. The GRADE certainty of evidence was low for the effectiveness of screening techniques and moderate to high for treatment efficacy. Antibody-detecting serological tests are the most effective screening tests for detection of both schistosomiasis and strongyloidiasis in low-endemicity settings, because they have higher sensitivity than conventional parasitological methods. Short courses of praziquantel and ivermectin were safe and highly effective and cost-effective in treating schistosomiasis and strongyloidiasis, respectively. Economic modelling suggests presumptive single-dose treatment of strongyloidiasis with ivermectin for all migrants is likely cost-effective, but feasibility of this strategy has yet to be demonstrated in clinical studies. The evidence supports screening and treatment for schistosomiasis and strongyloidiasis in migrants from endemic countries, to reduce morbidity and mortality.

## 1. Introduction

The public health importance of schistosomiasis and strongyloidiasis has increased in non-endemic regions as a result of growing global migration [1,2]. Schistosomiasis is caused by species of the trematode *Schistosoma* spp. *Sc. mansoni* is the most prevalent in Africa, the Americas, the Middle East and the West Indies, followed by *Sc. haematobium* in Africa and the Middle East and *Sc. japonicum* in east and south-east Asia [3]. Sub-Saharan African countries account for 90% of reported cases globally [3]. Prevalence rates of 10–50% for *Sc. haematobium* infections have been reported in some countries in sub-Saharan Africa and the Middle East [4], and prevalence rates of 1–40% have been reported for *Sc. mansoni* in sub-Saharan Africa and South America and for *Sc. japonicum* in Indonesia, parts of China and south-east Asia [5].

Strongyloidiasis is caused by the nematode *Strongyloides stercoralis* and, although it generally occurs in sub-tropical and tropical countries, it can be present in temperate countries where conditions are favourable [6]. The global burden of both diseases has been underestimated because of the poor sensitivity of diagnostic methods used in low-resource settings [6], but recent estimates indicate that around 370 million people are infected with *St. stercoralis* [7] and more than 200 million are infected with schistosomiasis causing a loss of more than 1.53 million disability-adjusted life years (DALYs) [4,5,8,9].

Few studies have assessed the prevalence schistosomiasis in European countries, but recent data show rates above 17% in migrants from sub-Saharan Africa [10]; prevalence of strongyloidiasis among refugee populations originating from south-east Asia and Africa was reported to be between 0.8% and 4.3% using microscopy; higher rates of between 9% and 77% using antibody detection assays were reported among refugees from south-east Asia [11]. Prevalence rates of 3.3%, 4.2% and 5.6% were reported in Italy, Spain and France, respectively, mainly in migrant populations or expatriates, without specifying diagnostic methods [6].

From all parasitic infections that may be highly prevalent among migrants, schistosomiasis and strongyloidiasis have several characteristics which support the rationale for screening based on the classical principles of Wilson and Jungner [12]. First, both infections are of particular importance, besides being as highly prevalent as other parasitic infections, they can cause long-term complications and severe consequences. Schistosomiasis is associated with chronic urogenital, hepato-intestinal and central nervous system complications [9,13,14,15]. *St. stercoralis* can cause disseminated infections or hyper infections with fatal outcomes in immunosuppressed patients (e.g., transplant recipients, those on corticosteroid therapy, with malignancies or co-infections with human T-cell lymphotropic virus-1 (HTLV-1)) [16]. In addition, there is a potential risk of transmission in the EU/EEA, either through organ transplantation in the case of strongyloidiasis [17] or through a favourable environment for the intermediate host, as in recent autochthonous cases of urinary schistosomiasis in Corsica, France which is not the case from many other parasitic infections [11,18]. Second, most infections are asymptomatic [13,19,20] and those infected are either unaware of their infection [19] or have very mild unspecific symptoms [3]. Third, both are chronic infections if untreated [19]. Schistosomiasis can remain as a sub-clinical infection for many years [3], and *St. stercoralis* replicates indefinitely inside the human host, causing lifelong infection if untreated [19].

Fourth, screening could be based on a simple and widely accessible technology, including commercially available serological test with a reasonable cost. In this sense, diagnosis of both infections based on microscopy has high specificity but low sensitivity [19,21,22]. Antibody-detecting serological tests offer higher sensitivity, at the expense of specificity, and have been shown to be useful in countries with low endemicity [19,22,23]. Finally, treatments for both infections are universally accepted with a high efficacy rate and low rate of adverse events. Praziquantel and ivermectin are the drugs of choice for treating schistosomiasis and strongyloidiasis, respectively [7,13].

In the last ten years, there has been a significant increase in migration patterns to the EU/EEA with some fluctuations in the volume and type of migration from year to year [24,25]. In 2017, migrants, here defined as being born abroad, made up 11% of this population, with 4% being born in another EU/EEA country and 7% originating from outside the EU/EEA [26]. There is an increased number of asylum applications with 56% of the 2,672,000 asylum decisions being positive between 2015 to 2017 [27]. Half of those denied asylum can be expected to leave, adding 580,000 to the EU/EEA’s total number of irregular migrants [28].

There is a notable gap in data collection on the disease burden, public health management, and in the surveillance for imported diseases in migrants arriving from endemic areas to EU/EEA. Geographic differences in disease distribution between global regions, influenced by increasing migration and population mobility from high endemic to non-endemic areas, remains an ongoing challenge to surveillance programmes and hampers the implementation of health policies concerning migrant health screening strategies [29,30]. 

There have been several systematic reviews addressing how effective are approaches to migrant screening infectious diseases in Europe [31,32,33], however parasitic infections are not adequately covered. Therefore, given the recent increase in migrants to the EU/EEA from endemic countries, there is a need for public health guidelines on the optimal approach to screening for schistosomiasis and strongyloidiasis [34,35,36]. In this systematic review, we assessed the effectiveness (and cost-effectiveness) of screening and management of these two parasitic infections in migrant populations. 

## 2. Methods

The review was one of six systematic reviews conducted under the auspices of a European Centre for Disease Prevention and Control (ECDC) project to develop guidance on screening for hepatitis C, hepatitis B, HIV, tuberculosis, vaccine-preventable diseases and parasitic infections in newly-arrived migrants to the EU/EEA [37]. The review group followed the Preferred Reporting Items for Systematic Reviews and Meta-Analyses (PRISMA) guidelines for the reporting of this systematic review [38]. The review protocol and methods assembled by a team of methodologists and clinicians with disease expertise was registered in Prospero (CRD42016045798) and published [39].

Our key research question was: 

What are the most effective screening and treatment options for schistosomiasis and strongyloidiasis in migrant populations arriving from endemic regions in the EU/EEA? 

To address this, we developed a logic model, prioritised outcomes important for the patient, and developed key questions along the evidence pathway (Appendix A). These key questions included:

(i) What are the best diagnostic tests to detect these infections non-endemic settings?
(ii) How effective are the drugs to treat them and what are the associated adverse events?
(iii) What are the most cost-effective screening and treatment options for schistosomiasis and strongyloidiasis in migrant populations from endemic regions in the EU/EEA?

### 2.1. Search Strategy and Selection Criteria

We searched for systematic reviews and meta-analyses in MEDLINE, Embase-ELSEVIER, the Cumulative Index to Nursing and Allied Health Literature (CINAHL), Epistemonikos, the Database of Abstracts of Reviews of Effects (DARE) and the Cochrane Database of Systematic Reviews (CDSR) for evidence on effectiveness. Our search used a combination of the key terms: ‘Immigrant’, ‘*Strongyloides*’, ‘Schistosomiasis’, ‘endemicity’, ‘prevalence’, ‘screening’, ‘migrant screening’, ‘mass screening’, ‘early detection’, ‘health impact assessment’ and ‘cost-effectiveness’ (Appendix B). The primary inclusion populations were migrants and refugees. We considered as main outcomes: cure, mortality, morbidity, adverse effects, health equity, quality of life and test accuracy measures (sensitivity and specificity). Also, we searched the National Health System (NHS) Economic Evaluation Database, the Health Economic Evaluations Database, the Cost Effectiveness Analysis Registry and Google Scholar for evidence on cost-effectiveness. We also identified any reviews on prevalence of the two infections. We restricted the search to studies published between 1 January 1993 and 30 May 2016. We did not apply language restrictions, and where we identified more than one version of a systematic review, we included the most recent. For the economic evidence, systematic reviews and primary studies of resource use, costs or cost-effectiveness of screening for schistosomiasis or strongyloidiasis with or without treatment were identified using specific search terms including (“costs and cost analysis”; “cost effectiveness analysis”; “costs.tw”; “cost$.mp”; “cost effective$.tw”; “cost-benefit analys$.mp” “health care costs.mp”) combined with clinical criteria. We reported all the costs in the local currency of the study setting or country, and in Euros using the Cochrane methods group purchasing power parity currency conversion calculator for the given year [40]. We also searched grey literature for published guidelines and reports on screening and prevention programme from the United States (U.S.) Centers for Disease Control and Prevention, ECDC, Joint United Nations Programme on HIV/AIDS (UNAIDS) and World Health Organization (WHO).

### 2.2. Additional Included Studies

Due to the limited evidence obtained from the initial search, we conducted an updated systematic search of six databases (MEDLINE, Embase-ELSEVIER, CINAHL, CDSR, DARE, Cochrane CENTRAL and Latin American Literature in Health Sciences—LILACS). We included relevant primary studies on diagnostic or screening tools for schistosomiasis (January 2010–February 2017) and strongyloidiasis (January 2012–February 2017). References of included primary studies were searched to identify other relevant studies.

### 2.3. Study Selection, Quality Assessment, and Synthesis

We included systematic reviews and evidence-based review guidelines which addressed each key question. When no systematic review was identified, we used primary studies. Two team members independently screened the titles and abstracts, followed by full-text assessments for eligibility of studies on prevalence, screening and treatment effectiveness, and related key questions (Eric Agbata, Nadia Montero) and of studies on cost-effectiveness (Nick Rowbotham, Rachael Morton). Disagreements were resolved by consensus or the involvement of a third author (AR). We assessed the methodological quality of reviews using AMSTAR [41] or Newcastle–Ottawa Scale [42] for reviews and observational studies respectively. We assessed the methodological quality of included primary studies on diagnostic effectiveness using the Quality Assessment of Diagnostic Accuracy Studies (QUADAS II) tool [43]. Synthesis of the studies and assessment of the certainty of the evidence for systematic reviews and individual studies was performed using GRADE (Grading of Recommendations Assessment, Development and Evaluation) methods, including Summary of Findings tables and Evidence to Decision tables [37]. For cost-effectiveness studies, we extracted the following data: economic study design (e.g., cost–utility analysis, Markov model), description of the case base population, the intervention and comparator, the absolute and relative difference in resource use and cost-effectiveness (e.g., incremental net benefit (INB) or incremental cost-effectiveness ratio (ICER).

## 3. Results

**Figure 1:**
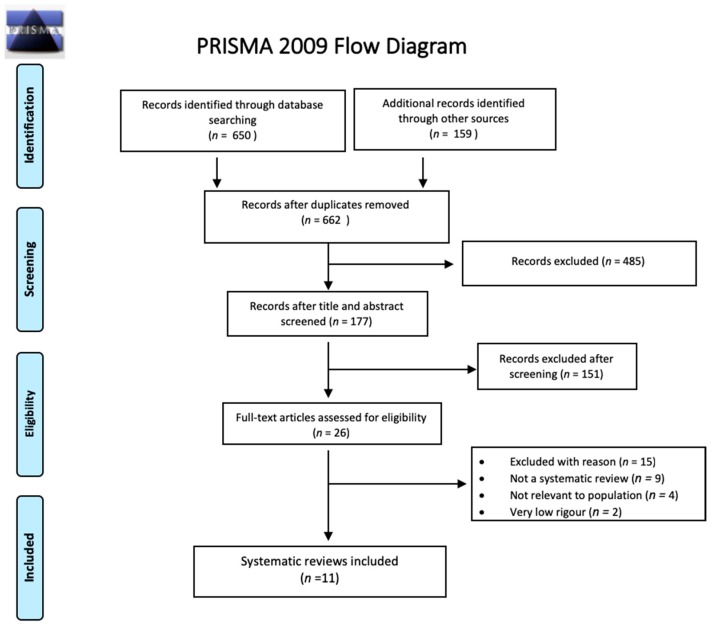
The Preferred Reporting Items for Systematic Reviews and Meta-Analyses (PRISMA) flow diagram for selection of systematic reviews on diagnostic accuracy and treatment efficacy for schistosomiasis and strongyloidiasis, (January 1993–May 2016).

**Figure 2:**
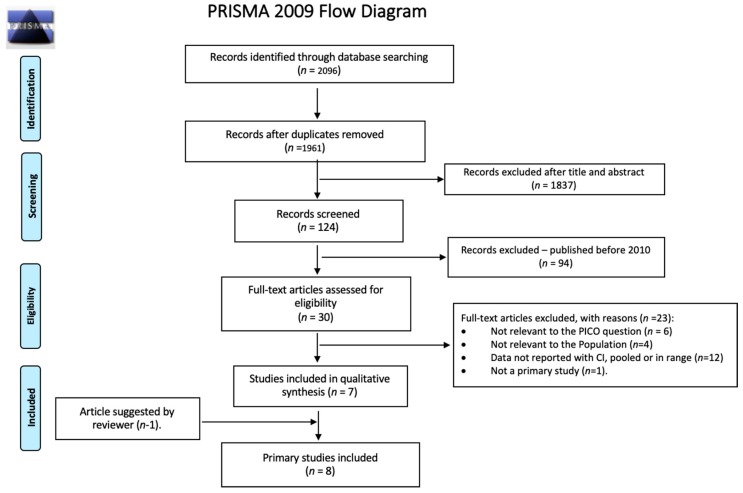
PRISMA flow diagram for selection of primary studies on diagnostic accuracy for schistosomiasis, January 2010–February 2017.

**Figure 3:**
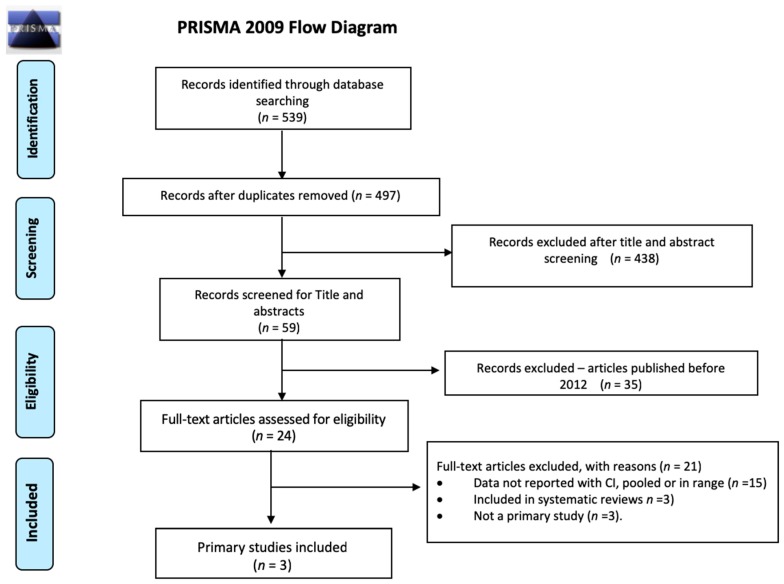
PRISMA flow diagram for selection of primary studies on diagnostic accuracy on strongyloidiasis, (January 2012–February 2017).

**Figure 4:**
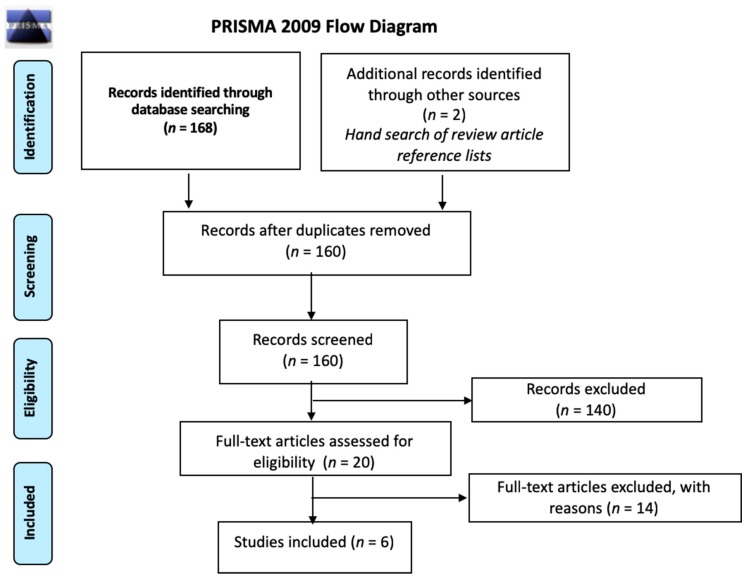
PRISMA flow diagram for selection of cost-effectiveness studies for schistosomiasis and strongyloidiasis, 1993–2016. DARE: Database of Abstracts of Reviews of Effects; NHS EED: National Health Service Economic Evaluation Database; Tufts CEA: Tufts Medical Centre Cost-Effectiveness Analysis Registry.

The first systematic search yielded, after removal of duplicates, 662 systematic reviews for which we screened titles and abstracts. Of the 26 systematic reviews selected for full-text screening, we included 11 systematic reviews which focused on the efficacy of diagnosis and treatment of schistosomiasis (*n* = 8) and strongyloidiasis (*n* = 3) (Figure 1) [19,44,45,46,47,48,49,50,51,52,53]. The updated systematic search for diagnostic testing accuracy studies for schistosomiasis yielded after de-duplication 1961 citations for the screening of titles and abstracts. Of the 30 articles selected for full-text screening, we included seven primary studies (Figure 2) [54,55,56,57,58,59,60]. One more primary research was identified later and included [61]. Another systematic search performed for diagnostic testing accuracy evidence for strongyloidiasis yielded 497 records after de-duplication; titles and abstracts were screened, and of the 24 papers selected for full-text screening, we included three primary studies (Figure 3) [62,63,64]. For the economic evidence, the search strategy yielded 160 studies after de-duplication. We retrieved 20 studies after title and abstract screening, of which six studies (four decision-analytic models for economic evaluation and two costing studies) were finally included—four for strongyloidiasis and two for schistosomiasis (Figure 4) [65,66,67,68,69,70]. Overall, we included 28 reviews and studies in this systematic review (Table 1, Table 2 and Table 3).

### 3.1. Screening: Diagnostic Test Accuracy for Schistosomiasis

**Figure 5:**
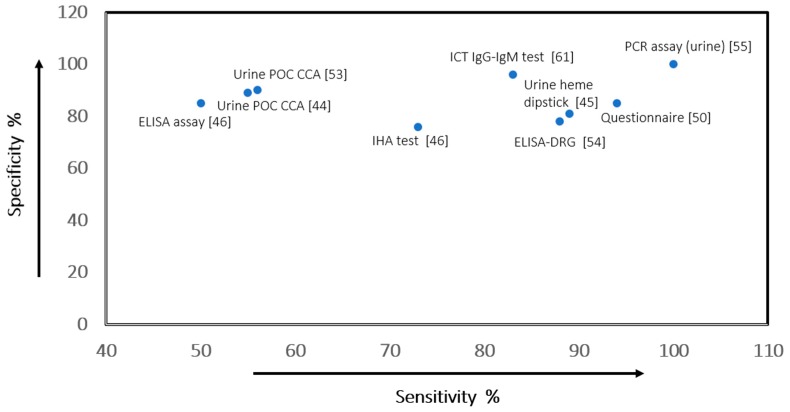
Scatter plot of sensitivity versus specificity values of the Index diagnostic tools for screening schistosomiasis.

We assessed diagnostic and screening tools for *Schistosoma* spp. in five included systematic reviews [44,45,46,50,53] and eight individual studies [54,55,56,57,58,59,60,61]. The best performing tests were included in the GRADE summary of finding on diagnostic tools for screening schistosomiasis (Table 4 and Figure 5).

#### 3.1.1. *Schistosoma Mansoni*

A meta-analysis reported estimated sensitivity and specificity values of 89% (95% CI: 86–92) and 55% (95% CI: 46–55) respectively, for the urinary circulating cathodic antigen (CCA) assay that detects *Sc. mansoni* in endemic areas [44]. Another urinary CCA test for *Sc. mansoni* [53] reported sensitivity and specificity values of 90% (95% CI: 84–94) and 56% (95% CI: 39–71), respectively compared with the duplicate Kato–Katz (KK) test (moderate-quality evidence) (Table 4). From the included primary studies, PCR assay in urine was the best-performing diagnostic test for *Sc. mansoni* with a sensitivity of 100% (95% CI: 95–100) compared with the CCA test—65% (95% CI: 56–77) and KK test—57% (95% CI: 46–68) [55] (very low-quality evidence); the specificity of PCR assay in urine was 100% (95% CI: 69–100) (Table 4) [55]. Espírito-Santo et al. reported sensitivity and specificity of 80% (95% CI: 28–99) and 92.4% (95% CI: 90–94), respectively for quantitative PCR (qPCR) in faeces compared with the KK test (not included in the GRADE Summary of findings) [56].

In low-endemic settings, the best-performing diagnostic test was the IgM-ELISA assay with sensitivity and specificity values of, respectively, 82% (95% CI: 64–93) and 82% (95% CI: 79–85)-low-quality evidence (Table 1) [57]. In another study, the ELISA-DRG kit showed the best accuracy with sensitivity and specificity values of, respectively, 78% (95% CI: 61–90) and 95% (95% CI: 89–98) (Table 4) [54]. In a recent study on the accuracy of different screening tests for schistosomiasis in African migrants, the immuno chromatographic test (ICT) IgG-IgM showed the best accuracy, with sensitivity and specificity values of 96% (95% CI: 91–99) and 83% (95% CI: 77–87) (Table 4) [61]. In all the individual studies, the certainty of evidence was very low to low.

#### 3.1.2. *Schistosoma Haematobium*

The urine heme dipsticks for the diagnosis of *Sc. haematobium* showed a mean sensitivity and specificity of 81% (95% CI: 73–83) and 89% (95% CI: 87–92), respectively, and were more accurate in high-prevalence than in low-prevalence settings -low-quality evidence (Table 4) [45]. Similarly, Ochodo et al. reported sensitivity and specificity values of 75% (95% CI: 71–79) and 87% (95% CI: 84–90)-low-quality evidence (Table 1) [44]. Furthermore, a meta-analysis on the diagnostic efficiency of questionnaire screening for schistosomiasis reported sensitivity and specificity values of 85% (95% CI: 84–86) and 94% (95% CI: 94–94) for *Sc. haematobium* infections (low-quality evidence) (Table 4) [50].

Kinkel et al. evaluated the accuracy of antibody-detection tests for diagnosis of imported *Sc. haematobium* [54]. The indirect haemagglutination (IHA) test with a sensitivity of 73% (95% CI: 56–86) and specificity of 99% (95% CI: 94–100) and the ELISA-DRG with a sensitivity of 78% (95% CI: 61–90) and specificity of 95% (95% CI: 89–98) demonstrated the best accuracy (certainty of evidence low) (Table 4) [54]. In another study, the ICT IgG-IgM test showed the best accuracy with sensitivity of 96% (95% CI: 91–99) and specificity of 83% (95% CI: 77–87) (Table 4) [61].

#### 3.1.3. *Schistosoma Japonicum*

In a meta-analysis of the accuracy of antibody detection of *Sc. japonicum* infection in humans, pooled sensitivities and specificities were 76% (95% CI: 74–77) and 73% (95% CI: 72–74) for the IHA test and 85% (95% CI: 83–87) and 50% (95% CI: 49–52) for ELISA (Table 4) [46]. 

The evidence also suggests that accuracy of diagnostic tests for schistosomiasis depends on pre-test prevalence (Table 5). As prevalence increased (from 2.5% to 30%), the estimated number of false-positives per 1000 migrants tested decreased with all tests—from 47 to 34 (*Sc. haematobium*/*Sc. mansoni*) [54], 58 to 42 (*Sc. haematobium*) [44], 107 to 77 (*Sc. Haematobium*) [45] and 166 to 119—(*Sc. haematobium*/*Sc. mansoni*) [61] per 1000 for ELISA-DRG, questionnaire screening, urine heme dipsticks and ICT IgG-IgM, respectively. The estimated false-negative tests were between 0–6 and 0–73 per 1000 at 2.5% and 30% prevalence for all the tests. At 2.5% pre-test prevalence, the proportion of correctly diagnosed schistosomiasis infections in migrant populations was 100% for the urine PCR assay, 96% for the ICT IgG-IgM test, 90% for the urine POC CCA, 85% for the questionnaire screening and 84.9% for *Sc. japonicum* ELISA (Table 5).

### 3.2. Screening: Diagnostic Test Accuracy for Strongyloidiasis

We assessed diagnostic and screening tools for *St. stercoralis* in two included systematic reviews [19,51] and three individual studies (Table 1 and Table 6) [62,63,64].

The best conventional diagnostic tools for *St. stercoralis* have been agar plate culture with a sensitivity and specificity of 89% (95% CI: 86–92) and 100% (95% CI: 100–100) respectively, and the Baermann method with a sensitivity and specificity of 72% (95% CI: 67–76) and 100% (95% CI: 100–100) respectively (moderate certainty of evidence) [51]. Knopps et al. reported a much lower sensitivity value of 31% (95% CI: 19.1–44.8) for PCR in stools compared with a combination of stool-based methods as the gold standard; specificity was 100% (95% CI: 100–100) [64].

**Figure 6:**
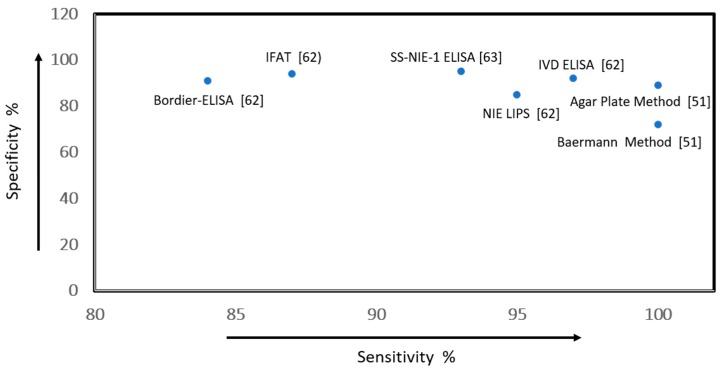
Scatter plot of sensitivity versus specificity values of the Index diagnostic tools for screening strongyloidiasis.

Serological antibody detection methods have demonstrated greater sensitivity compared with classical parasitological techniques [19]. Bisoffi et al. reported the accuracy of five serological tests for detection of strongyloidiasis [62]. The sensitivity and specificity values were: 85% (95% CI: 79–92) and 100% (95% CI: 100–100) for the luciferase-immunoprecipitation system (LIPS) using 31-kD recombinants antigen from *St. stercoralis* (NIE); 75% (95% CI: 66–83) and 95% (95% CI: 91–99) for the NIE-ELISA (using the same antigen); 91% (95% CI: 86–96) and 99% (95% CI: 97–100) for the IVD-ELISA; 90% (95% CI: 84–95) and 98% (95% CI: 96–100) for the Bordier-ELISA; and 94% (95% CI: 90–98) and 92% (95% CI: 87–97) for the indirect fluorescent antibody technique (IFAT) (low certainty of evidence) [62] (Figure 6). Rascoe et al. reported comparable values for two new recombinant antigens in antibody detection assays: SS-NIE-1 ELISA with sensitivity of 95% (95% CI: 92–97) and specificity of 93% (95% CI: 90–96), and Ss-NIE-1 Luminex with sensitivity of 93% (95% CI: 86–96) and specificity of 95% (95% CI: 93–97) (Table 6) [63].

As with schistosomiasis, estimates of false-positive tests per 1000 tested decreased with increasing pre-test prevalence, from 29 to 21, 58 to 42 and 68 to 49 for IVD-ELISA, Bordier-ELISA and SS-NIE-1 ELISA assays, respectively [62,63]. The estimated number of false-positive tests for the Baermann and Agar plate methods was 0 at all pre-test prevalence levels. Lower numbers of false-negatives were estimated for all the serological tests, for example, 1 and 15, and 2 and 24, per 1000 tests for SS-NIE-1 and IVD-ELISA at 2.5% and 30% prevalence levels compared with 3 and 33, and 7 and 84, per 1000 for the Agar plate and Baermann methods. At 2.5% pre-test prevalence, the proportion of correctly diagnosed *Strongyloides* infections in migrant populations was 95% for the SS-NIE-1 ELISA, 93.8% for IFAT, 92% for IVD-ELISA and 90.7% for Bordier-ELISA, compared with 72% and 89% for the Baermann and Agar plate methods (Table 7).

### 3.3. Treatment Efficacy: Schistosomiasis and Strongyloidiasis

We evaluated four included systematic reviews on treatment of schistosomiasis and strongyloidiasis (Table 8 and Table 9) [47,48,49,52]. In a Cochrane review, the efficacy of praziquantel (single 40 mg/kg dose) showed much lower parasitological failure in urine (<53%) at 1 to 2 months (RR = 0.42; 95% CI: 0.29–0.58) compared with placebo [48]. The proportion of people cured with praziquantel varied substantially between trials, from 22.5% to 83.3%, but was higher than 60% in five of the seven trials [48]. Similarly, in another Cochrane review, parasitological cure rate for *Sc. mansoni* infection at one month with praziquantel (single 40 mg/kg dose) varied substantially across studies, ranging from 52% to 92% in Brazil in 2006 and 2007, for example parasitological cure 66% more in intervention group compared with placebo (RR 3.13; 95% CI: 1.03–9.53) (Table 8) [47]. Pérez del Villar et al. compared the efficacy of praziquantel and artemisinin derivatives and reported that artesunate showed significantly lower cure rates than praziquantel 30% vs. 61% (RR 0.49 (0.28–0.75)) [49]. Artemeter monotherapy (6mg/kg single dose) reduced *Sc. Japonicum* infection rates in patients (RR = 0.25; 95% CI: 0.16–0.40). However, a combination of artemisinin derivatives plus praziquantel showed higher cure rates than praziquantel monotherapy (RR = 1.25; 95% CI: 1.09–1.37) in areas with intense transmission (moderate certainty of evidence) (Table 8) [49]. No significant adverse events were reported. 

Only one systematic review was included which addressed the efficacy of ivermectin vs. albendazole or thiabendazole for treating chronic strongyloidiasis infection (Table 9) [52]. Parasitological cure determined with both serological and conventional techniques was higher with ivermectin (single-/double-dose) treatment than with albendazole 84% vs. 48% (RR = 1.79; 95% CI: 1.55–2.08) (moderate-quality evidence) [52]. When ivermectin was compared with thiabendazole, there was no distinction in parasitological cure, i.e., 74% vs. 68% (RR = 1.07; 95% CI: 0.96–1.2), but adverse events were less frequent with ivermectin (RR = 0.31; 95% CI: 0.20–0.50) than with thiabendazole [52] (moderate certainty of evidence). No serious adverse events or deaths were reported with either ivermectin or thiabendazole.

### 3.4. Resource use, Costs and Cost-Effectiveness

#### 3.4.1. Strongyloidiasis

Three economic studies of moderate quality support a strategy of presumptive treatment for strongyloidiasis in migrants from high-risk backgrounds [66,67,68]. One study showed potential cost savings of universal treatment with albendazole compared with i) no intervention (watchful waiting); and compared with ii) universal stool-based screening; in migrant populations in the U.S. [66]. Sensitivity analyses indicated a best-case scenario of large savings from presumptive treatment, and a worst-case scenario in which treatment was still cost effective at the $30,000/QALY threshold (1997 U.S. dollars).

The second study on presumptive treatment for strongyloidiasis in migrants living in the U.S. in California and New York compared: i) presumptive treatment with albendazole for 3 or 5 days; ii) presumptive treatment with one dose of ivermectin; iii) treatment in those with documented eosinophilia; and iv) no intervention [67]. It indicated that presumptive treatment with ivermectin was cost-effective at a threshold of less than USD 10,000 (EUR 9667) per QALY across a range of prevalence values in migrants living in the U.S. [67]. This study did not include antibody detection among the diagnostic tools. At a prevalence higher than 10%, treatment with ivermectin cost less than USD 2000 (EUR 1983) per QALY. These results were robust across a wide range of sensitivity analyses [67].

The third more recent study on presumptive treatment for hookworm and strongyloidiasis in U.S.-bound Asian populations indicated that treatment in the destination country with albendazole and ivermectin was likely to be cost-effective relative to no screening or screening and treatment strategies in the country of origin among refugees from high-prevalence countries [68]. For strongyloidiasis, overseas treatment cost less than USD 40,000 (EUR 31,092) per QALY gained at prevalence greater than 1% and fell to less than USD 18,000 (EUR 13,991) per QALY gained at prevalence greater than 3%.

#### 3.4.2. Schistosomiasis

There were no cost-effectiveness studies of screening and presumptive treatment in migrants at risk of schistosomiasis. In non-migrant populations, a recent costing study compared the costs of single and double KK tests with a urine dipstick test [69] for *Sc. haematobium* diagnosis in areas of high endemicity. The results of this preliminary costing study indicated similar costs of around USD 6–7 (EUR 5–6) per test for single KK stool and urine tests; however, the quality of evidence for resource use was low. A cost-effectiveness study by King et al. compared single-dose (40 mg/kg body weight) and double-dose (40 mg/kg doses separated by 2–8 weeks) presumptive treatment with praziquantel for schistosomiasis in high-prevalence (>40%) settings in Africa [65]. Double-dose praziquantel was found deemed to be highly cost-effective (ICER of less than USD 500 (EUR 471)/QALY) compared with single-dose treatment.

## 4. Discussion

The rationale for screening for strongyloidiasis and schistosomiasis in the EU/EEA and not other parasitic infections is based on the estimated prevalence of these parasitic infections among migrants from endemic countries; potential prevention of fatal complications through early case detection and treatment, and secondary transmission in asymptomatic patients based on a highly sensitive test and very effective and safe treatment [11,35,36,71]. Therefore, the implementation of a screening programme would allow early detection of the infection in individuals at risk, before they develop a severe condition which may justify the screening itself.

Although quality data on the prevalence of schistosomiasis and strongyloidiasis among migrant populations in the EU/EEA is limited, available data from endemic regions shows that prevalence of schistosomiasis is between 20% and 40% and prevalence of strongyloidiasis is between 10% and 40% [3,4,5]. However, there is a rationale for public health surveillance for schistosomiasis and strongyloidiasis to inform proper surveillance of mobile population from the regions [30]

Overall, systematic reviews showed that antibody-detecting serological tests are the most effective screening tests for detection of schistosomiasis and strongyloidiasis in low-endemicity settings, because they have higher sensitivity than conventional parasitological methods [19,44,45,50,53]. Newer serological tests were shown to be more effective than conventional techniques such as agar plate culture and the Baermann method for strongyloidiasis and KK for *Sc. mansoni*. These conventional techniques, as well as PCR, failed to detect infections of very low intensity [64] although they were more specific than serological techniques [51,54]. They are also labor-intensive and require skilled personnel and are therefore not recommended as the first option for screening [19]. In contrast, serological testing is easier to perform in health facilities than collecting and testing faecal samples and can also be combined with other infectious disease screening tests. 

One limitation of antibody-detecting serological tests, particularly with schistosomiasis, is that they cannot differentiate current from past infections; however, with strongyloidiasis, antibody titres decline after treatment over time in most patients [62,72]. In addition, in immuno-compromised patients, the sensitivity of serological tests may be reduced, and other additional screening methods may be needed if serology is negative. In this regard, the utility of PCR assay as an alternative screening method in immunosuppressed patients deserves further investigation.

Specifically, for *Schistosoma* spp. infections, available evidence shows that the IgM-ELISA [57], IHA [46] and ICT IgG-IgM [61] tests were the most effective screening tests in low-endemicity countries. In some low endemicity settings, two serological tests are performed, and a case is considered to be positive if either test is positive; in others, a combination of ELISA testing and KK faecal examinations is used to improve the accuracy of detection. However, Beltrame et al. advocate the use of the ICT IgG-IgM test as a single screening test (negative predictive value >97%) [61].

For strongyloidiasis, available evidence (of very low to low quality) shows that antibody-detecting blood tests using a variety of antigen preparations have a better detection rate than conventional parasitological methods, with IVD-ELISA, Bordier-ELISA and NIE LIPS being the most accurate tests [62]. Limitations of these serological tests include the large number of infective larvae required, cross–reactions with other nematode infections and lower sensitivity in immuno-compromised patients [19,62]. New tests based on the recombinant antigen Ss-NIE-1, although slightly less sensitive, but currently considerably more expensive than other serological techniques, show excellent specificity [62,63] and, although not widely available, they may be useful when designing rapid tests [63].

For treatment of schistosomiasis, single-dose praziquantel is the drug of choice. Evidence from systematic reviews shows that treatment with praziquantel significantly increased parasitological cure and, achieved marked reductions in microhaematuria compared with placebo; praziquantel also has a very good safety profile [47,48]. For treatment of strongyloidiasis, there is evidence (of low to moderate quality) that ivermectin is more effective than albendazole [52] and evidence (of moderate quality) that ivermectin is as effective as thiabendazole, but much better tolerated; no difference in the efficacy of ivermectin was observed between endemic and non-endemic populations [52]. However, there are no studies on the potential harms of large-scale administration of ivermectin (although widespread experience with filariasis control is reassuring). 

Implementing presumptive treatment either with ivermectin or praziquantel requires additional complex screening strategies to identify individuals with loiasis or neurocysticercosis for whom these drugs might be inappropriate [70,71] and recently published recommendations specify that immigrants arriving from endemic areas should undergo a thorough clinical screening before being given either praziquantel or albendazole [73]. In addition, ivermectin is not readily available in most endemic and non-endemic countries and has limited approval by regulatory authorities in the EU/EEA.

We found no studies evaluating the cost-effectiveness of schistosomiasis screening and treatment interventions in migrant populations. For schistosomiasis, no studies were available on the cost of screening tests based on antibody detection in the non-endemic setting. In endemic settings, double-dose praziquantel was deemed to be highly cost-effective compared with a single dose and was considered robust to plausible changes in parameter estimates [65]. Further economic studies are required to provide better data on the cost-effectiveness of a test-and-treat strategy for schistosomiasis in non-endemic countries. For strongyloidiasis, three studies indicated that presumptive treatment with albendazole or ivermectin was cost-saving or cost-effective, in migrants to the U.S. or in endemic settings [66,67,68]. The limitations of these studies may decrease the relevance of the results for migrant populations in the EU/EEA. Most of the economic studies identified were limited to Asian populations and not based on screening with antibody testing in a non-endemic setting. However, where the prevalence of schistosomiasis and strongyloidiasis is greater than 1% and the price of presumptive treatment is similar to that used in the economic evaluations identified in this review, presumptive treatment with ivermectin or albendazole is likely to be cost-effective for migrants to the EU/EEA.

The strengths of our study include the use of the GRADE methodology to evaluate the quality and strength of the evidence and effect size in the included studies. The primary outcomes—parasitological cure or failure for efficacy of treatment and accuracy for screening—were objective measures. The individual studies in the included systematic reviews originated from different regions and countries with moderate to high endemicity for both parasites, increasing the generalizability of the results.

We did not identify any systematic reviews or RCTs on screening for schistosomiasis and strongyloidiasis in newly arrived migrants to EU/EEA. RCTs on preventive screening are rare, and so we used a logic model approach, as recommended at US Task Force on Preventive Health Care, and present data on population prevalence, diagnostic accuracy, treatment effectiveness and cost-effectiveness [70,74]. Other limitations include the lack of accurate data on the prevalence of schistosomiasis and strongyloidiasis among migrants from endemic countries entering the EU/EEA and the lack of data on the cost-effectiveness of screening and treating migrants for these parasitic infections. Further studies evaluating the effectiveness and cost-effectiveness of screening intervention in migrant populations are warranted.

The results of this systematic review indicate that although the certainty of desirable over undesirable effects of screening mobile and high-risk migrant populations from endemic areas is low to moderate, there is a rationale for screening, particularly in immunosuppressed patients since there is a high value placed on uncertain but potentially life-preserving benefits as suggested elsewhere [75]. Both schistosomiasis and strongyloidiasis can become chronic and cause severe long-term complications if untreated and the health benefits of intervention therefore outweigh its potential harms. Effective diagnostic tests are available and treatments for both infections are efficacious, well tolerated and safe with few exceptions [48,52,54,62]. 

Presumptive single-dose therapy of strongyloidiasis with ivermectin for all migrants is likely to be cost-effective; however, the feasibility of this measure has not been demonstrated in clinical studies in non-endemic settings. Importantly, implementing presumptive treatment either with ivermectin for strongyloidiasis or praziquantel for schistosomiasis requires additional screening strategies to identify individuals for whom these drugs might be harmful.

The evidence suggest screening should target people arriving from endemic areas, but national screening strategies will need to be tailored to the specific context of individual EU/EEA countries and, in particular, the countries of origin of migrants to those countries. Although, there are no studies on the extent to which multiple screening tests for infectious diseases in migrants can improve cost-effectiveness, integrating innovative public health screening strategies for schistosomiasis and strongyloidiasis with other infectious diseases will improve surveillance data as well as reduce costs. 

However, the optimal approach to delivery of screening will need to consider a global perspective, as well as depend on the health system context in individual EU/EEA countries. In this regard, addressing lack of access to healthcare for migrants, heterogeneity of screening strategies applicable in member states, and improving health professionals’ knowledge and training of migrant related infectious diseases should improve the responsiveness of the public health care system with regards to coverage and uptake of screening at the level of primary health care. 

Finally, although we consider that sufficient evidence exists to justify screening for strongyloidiasis and schistosomiasis immigrants coming to the EU/EEA from endemic areas, further assessment of the benefits and risks of screening and treatment is needed. More specifically, additional economic analysis is required, in particular to evaluate the costs of a test and treat strategy and to compare the cost-effectiveness of screening and of presumptive treatment.

## 5. Conclusions

This systematic review provides a compendium of indirect evidence that support the screening for strongyloidiasis and schistosomiasis in migrants coming from endemic areas to the EU/EEA, and particularly in immunosuppressed or at-risk-of immunosuppression patients. 

Screening for strongyloidiasis and schistosomiasis should be considered based on serological testing in the absence of immunosuppression. Ivermectin and praziquantel have demonstrated a high efficacy, an excellent safety profile, and a potentially easy schedule for the treatment of strongyloidiasis and schistosomiasis. Economic modelling suggests presumptive single-dose treatment of strongyloidiasis with ivermectin for all migrants is likely cost-effective, but the feasibility of this strategy has yet to be demonstrated in clinical studies in non-endemic settings.

## Figures and Tables

**Table 1:** Characteristics of included studies on diagnostic test effectiveness for schistosomiasis and strongyloidiasis, January 1993–February 2017.

Study	Quality	Design	Population	Intervention/Outcomes	Results
Included systematic reviews of diagnostic tests to detect schistosomiasis					
Danso Appiah et al., 2016 [53]	AMSTAR: 11/11 GRADE: low to moderate-quality evidence	Systematic review and meta-analysis	Preschool children and infants, school-aged children or adults from high-/low-prevalence locations	Intervention: POC CCA for *Sc. mansoni* Outcomes: detection of egg-positive urine—sensitivity/specificity (95% CI:)	Sensitivity/specificity (95% CI) POC CCA (single standard) 90% (84–94)/56% (39–71); POC CCA (duplicate standard) 85% (80–88)/66% (53–76); POC CCA (triplicate standard) 91% (84–95)/56% (39–72)
Yang et al., 2015 [50]	AMSTAR: 11/11 GRADE: low to moderate-quality evidence	Meta-analysis	Patients infected with schistosomiasis in endemic areas; mainly school children, Africa and China	Intervention: questionnaire screening for Schistosoma species. Outcomes: sensitivity/specificity (95% CI:)	Sensitivity/specificity (95%CI:) *Sc. haematobium* 85% (84–86)/94% (94–94); *Sc. mansoni* 46% (45–47)/81% (80–82); *Sc. japonicum* 82% (79–85)/59% (57–60)
Ochodo et al., 2015 [44]	AMSTAR: 11/11 GRADE: very low to low-quality evidence	Systematic review and meta-analysis of RCTs	Individuals with active infection with S. haematobium	Intervention: urine reagent strip tests; circulating antigen tests in urine/serum Outcomes: sensitivity/specificity (95% CI:)	Sensitivity/specificity (95% CI) *Sc. haematobuim*: microhaematuria 75% (71–79)/87% (84–90); proteinuria 61% (53–68)/82% (77–88); leukocyturia 58% (44–71)/61% (34–88); *Sc. mansoni* (CCA test) 89% (86–92)/55% (46–65)
King and Bertsch, 2013 [45]	AMSTAR: 11/11 GRADE: low-quality evidence	Systematic review and meta-analysis of surveys	Schools, communities with high/low prevalence, low intensity groups in Africa	Intervention: dipstick test *Sc. haematobium*. Outcomes: sensitivity and specificity (95% CI:), diagnostic odds ratio (DOR)	Sensitivity/specificity (95% CI) Detection of egg-positive urine 81% (79–83)/89% (87–92). In high-prevalence settings 80% (78–83)/86% (82–90); lower in treated population 72% (61–78)/87% (81–94); in lower intensity population subgroups 65% (58–72)/82% (76–90)
Wang, et al., 2012 [46]	AMSTAR: 7/11 GRADE: very low- to low-quality evidence	Systematic review and meta-analysis of RCTs, retro-/pro-observational studies	Infected patients with schistosomiasis in control programmes in China	Intervention: IHA and ELISA. Outcomes: true positive rates, sensitivity/specificity (95% CI:), DOR	Sensitivity/specificity (95% CI) IHA 75.6% (74–77)/73% (72–74) ELISA 84.9% (83–87)/50.4% (49.2–51.6) The DOR of IHA was 9.41 (95% CI: 5–18), and ELISA 4.78 (95% CI: 3.21–7.13)
Included primary studies of diagnostic tests to detect schistosomiasis					
Espirito-Santo et al., 2015 [57]	QUADAS-2-11/14 GRADE: very low- to low-quality evidence	Cross-sectional epidemiological survey in areas of low prevalence of *Sc. Mansoni*	The estimated sample size required was 650 individuals; Barra Mansa City, Rio de Janeiro State, Brazil	Intervention: diagnostic assays: ELISA-IgG/ELISA-IgM/IFT-IgM/qPCR in faeces. Outcomes: sensitivity/specificity (95% CI:)	Sensitivity/specificity (95% CI) KK 13.8% (4–32)/99.8% (99.0–100); ELISA-IgG 66.7% (48–82)/91.5% (89–94); ELISA-IgM 81.8% (64–93)/82% (79–85); IFT-IgM 78.8% (61–91)/87.7% (84.8–90); qPCR in faeces 51.7% (32–71)/92.6% (90–95); qPCR in serum 12.1% (3–28)/99.1% (98–99)
Espirito-Santo et al., 2014a [60]	QUADAS-2-12/14 GRADE: very low- to low-quality evidence	Cross-sectional study	City of Barra Mansa, Rio de Janeiro State, Brazil, with an estimated prevalence of 1%	Intervention: diagnostic assays: ELISA-IgG and ELISA-IgM. Outcomes: sensitivity/specificity (95% CI:); PPV, NPV	Sensitivity/specificity (95%CI) ELISA-IgG 60.0% (15–95) /89.1% (86.2–91.5); ELISA-IgM 60.0(15–95)/79.2% (75.6–82.5) PPV/NPV (95%CI): ELISA-IgG 4.6% (1–13) /99.6% (98–100); ELISA-IgM 2.5% (0.5–7); NPV 99.6% (98.4–100.0)
Espirito-Santo et al., 2014b [56]	QUADAS-2-13/14 GRADE: very low- to low-quality evidence	Cross-sectional epidemiological survey	7000 inhabitants located in the outskirts of Barra Mansa, Rio de Janeiro, Brazil	Intervention: qPCR in serum or faeces. Outcomes: sensitivity/specificity (95% CI:); PPV, NPV	Sensitivity/specificity (95% CI) qPCR in faeces 80.0% (28–99)/92.4% (90–94); qPCR in serum 20.0% (0.5–71.6)/98.8 (97–99) PPV/NPV (95% CI:): qPCR in faeces 8.0% (2–19)/99.8% (99–100); qPCR in serum 12.5% (0.3–52.7)/99.3% (98.2–99.8)
Lodh et al., 2013 [55]	QUADAS-2-12/14 GRADE: very low- to low-quality evidence	Cross-sectional case study	Filtered urine specimens from infected and not-infected patients in Zambia	Intervention: qPCR ELISA IgG in serum or faeces; filtered Urine PCR. Outcomes: sensitivity/specificity (95% CI:); PPV, NPV	Sensitivity/specificity (95%CI) KK test 57% (45–68)/100% (69–100); CCA rapid test 65% (56–77)/60% (26–88); PCR 100% (95–100)/100% (69–100) PPV/NPV: KK test 100%/23%; CCA rapid test 93%/19%; PCR 100%/100%.
Kinkel et al., 2012 [54]	QUADAS-2-12/14 GRADE: very low- to low-quality evidence	Retrospective comparative diagnostic study: performance of 8 serological tests for Schistosoma spp	Serum specimens from infected patients and those without the infection in low-prevalence locations or non-endemic settings (Germany)	Intervention: serological assays: IFAT, ELISA-CA, ELISA-AWA, ELISA-SEA, IHA, ELISA-NovaTec, ELISA-DRG and ELISA-Viramed. Outcomes: sensitivity and specificity (95% CI:)	Sensitivity/specificity-(95% CI): IFAT 75.7% (58–98)/98.1% (92–99); ELISA-CA 40.5% (25–59)/95.2% (89–98); ELISA-AWA 54.1% (37–70)/100% (95.6–100); ELISA-SEA-75.7% (58–98)/97.1% (91–99); IHA 73.0% (55.6–85.6)/99.0% (94.0–100); ELISA-NovaTec 64.9% (47–79)/99 (94–100); ELISA-DRG 78.3% (61.3–89.6)/88.4 (80–94); ELISA-Viramed 67.6% (50–81)/76.9% (67–84).
De Frotas et al., 2011 [58]	QUADAS-2-12/14 GRADE: very low- to low-quality evidence	Cross-sectional survey	Stool and serum specimens from infected and not infected patients, low-endemic setting in Brazil	Intervention: serological assays, ELISA IgG Outcomes: sensitivity and specificity (95%CI)	Sensitivity/specificity (95% CI): ELISA-IgG 100% (68–100)/72.9% (67–78). PPV/NPV (95% CI): ELISA-IgG 26.0% (18–36) /100% (97–100).
Silveira et al., 2016 [59]	QUADAS-2-12/14 GRADE: very low- to low-quality evidence	Evaluation of the CCA test to diagnose *Sc. mansoni* in Minas Gerais State, Brazil.	Infected individuals in regions with moderate to high prevalence	Intervention: CCA-immuno-chromatographic test. Outcomes: sensitivity/specificity (95% CI:)	Sensitivity/specificity (95% CI): CCA-ICT 68.7% (54–81)/97.6% (87–99)
Beltrame et al., 2017 [61]	QUADAS-2-12/14 GRADE: very low- to low-quality evidence	Accuracy of parasitological and immunological tests for the screening of human schistosomiasis in immigrants and refugees from African countries	Frozen serum specimens from recent African asylum seekers that were routinely screened for schistosomiasis in Italy	Intervention: urine CCA; Bordier-ELISA, Western Blot IgG, ICT IgG-IgM, microscopy compared with composite reference standard. Outcomes: sensitivity/specificity (95% CI:)	Sensitivity/specificity (95% CI): Urine CCA 29% (22–37)/95% (91–97); Bordier-ELISA 71% (63–78)/99.6% (98–100); Western blot IgG 92% (86–96)/94% (90–97); ICT IgG-IgM 96% (91–99)/83% (77–87); microscopy 45% (37–54)/100%
Included systematic reviews for diagnostic effectiveness for strongyloidiasis					
Campo Polanco et al., 2014 [51]	AMSTAR: 11/11 GRADE: moderate-quality evidence	Systematic review and meta-analysis	Individuals with active/chronic infection	Intervention: Baermann method, agar plate, direct faecal smear examination and formol-ether concentration technique. Outcome: sensitivity and specificity (95% CI:)	Sensitivity: Baermann method (72%) with LR+228 and LR−0.32; APC 89%, LR+341 and LR−0.11; stool microscopy 21%, LR + 67 and LR−0.67; formol-ether concentration 48%, LR + 110 and LR−0.59. Specificity: 100% in all four tests. APC and Baermann method are best.
Requena-Méndez et al., [19]	AMSTAR: 7/11 GRADE: low- to moderate-quality evidence	Systematic review	Individuals with active/chronic infection	Intervention: Baermann method, agar plate, direct faecal smear examination and formol-ether concentration technique, serological techniques. Outcome: sensitivity and specificity (95% CI:)	No meta-analysis was undertaken. Sensitivity and specificity of different techniques were individually reported.
Included primary studies for diagnostic effectiveness for strongyloidiasis					
Bisofi et al., 2014 [62]	QUADAS-2: 13/14 GRADE: low-quality evidence	Retrospective comparative diagnostic study to evaluate the performance of 5 tests for *St. stercoralis.*	Serum specimens from subjects with *St. stercoralis*; healthy people and patients with previous exposure	Intervention: IFAT, NIE-LIPS NIE-ELISA, IVD-ELISA- and Bordier-ELISA Outcome: sensitivity and specificity (95% CI:)	Sensitivity/specificity (95% CI): NIE-ELISA 75.4% (67–83)/94.8% (91–99); NIE-LIPS 85.1% (78–92)/100% (100–100); IFAT 93.9% (89–98)/92.2% (87–97); IVD-ELISA 91.2% (86–96)/99.1% (97.4–100.0); Bordier-ELISA 89.5% (84–95) 98.3% (96–100).
Rascoe et al., 2015 [63]	QUADAS-2: 10/14 GRADE: low-quality evidence	Retrospective comparative diagnostic study of 5 tests for the follow-up of patients infected with *St. stercoralis*	Serum samples positive for *St. stercoralis* and negative samples from United States residents with no history of foreign travel	Intervention: Ss-NIE-1 ELISA, Ss-NIE-1 Luminex. Outcome: sensitivity and specificity (95% CI:)	Sensitivity/specificity (95% CI): SS-NIE-1 ELISA 95% (92–97)/93% (90–96); Ss-NIE-1 Luminex 93% (88–96)/95% (93–97). The inter-assay coefficient of variation was determined to be 22% for the low-positive control serum and 10% for the medium-positive control serum.
Knopp et al., 2014 [64]	QUADAS-2: 11/14 GRADE: low-quality evidence	International standard randomised controlled trial	Children and adults residing in rural villages in the Baga moyo District, Tanzania (endemic areas)	Intervention: Real-time PCR, FLOTAC technique, KK method. Outcome: sensitivity and specificity (95% CI:)	Sensitivity/specificity (95% CI): PCR + pseudo-standard PCR 17.4 (8–31)/3.9 (89–97); Baermann + pseudo-standard 47 (23–72)/78.4 (72 -84); PCR + multiple gold standard 30.9 (19.1–44.8)/100 (100–100); Baermann + multiple gold standard 83.6 (71.2–92.2)/100 (100–100)

AWA: adult worm antigen; AMSTAR: a tool for assessing the methodological quality of systematic reviews; APC: agar plate culture; CA: Cercarial antigen; CCA: circulatory cathodic antigen; CI: confidence interval; DOR: diagnostic odds ratio; GRADE: Grading of Recommendations, Assessment, Development and Evaluation; ELISA: enzyme-linked immunosorbent assay; FLOTAC: novel multivalent faecal egg count method; ICT: Immuno chromatographic test; IFAT: indirect fluorescent antibody technique; IHA: indirect haemagglutination: In Vitro Diagnostic kit; KK: Kato–Katz method; LIPS: luciferase immunoprecipitation system; LR+: positive likelihood ratio; LR−: negative likelihood ratio; NIE: a 31-kDa recombinant antigen; NovaTec: NovaTec Immundiagnostica, Dietzenbach, Germany; NPV: negative predictive value; POC: point-of-care; qPCR: quantitative PCR (real-time polymerase chain reaction); PPV: positive predictive value; RCT: randomised controlled trial; SEA: soluble egg antigen; Ss-NIE-1: a luciferase tagged recombinant protein of *St. stercoralis* for IgG and IgG_4_ specific antibodies; QUADAS-2: a tool for the quality assessment of diagnostic accuracy studies; Viramed^®^: Viramed Biotech, Planegg, Germany).

**Table 2:** Characteristics of included studies about efficacy of treatment for schistosomiasis and strongyloidiasis, 1993–2016.

Study	Quality	Design	Population	Intervention/Outcomes	Results
Treatment efficacy of anti-Schistosoma drugs					
Kramer et al., 2014 [48]	AMSTAR: 11/11 Data in study: GRADE: high-quality evidence	Systematic review, fixed effects meta-analysis; Embase, MEDLINE (1966 to 2014), LILACS, Cochrane library, Cochrane infectious disease (1980–2014)	School-aged and young adults: 6–20 years (16 trials); 2–23 years (5 trials); Adults (2 trials). Participants setting: Rural areas in 15 sub-Saharan African countries; an urban setting in Saudi Arabia	Interventions: drugs used to treat urinary schistosomiasis: praziquantel, metrifonate, artesunate and/or in combination Outcome: parasitological cure or failure at 4 weeks; % egg reduction rate at 4 weeks	Praziquantel (single dose 40 mg/kg), egg reduction (60%) in urine achieved in 4–8 weeks (38 per 100 (95% CI: 26–54). Treatment failure: RR 0.42, (95% CI: 0.29–0.59), 864 participants, 7 trials Metrifonate (single dose 10 mg/kg) reduced egg excretion only marginally in comparison to placebo (RR 0.63, 95% CI: 0.54 to 0.73) 210 participants, 1 trial, at 8 months
Danso-Appiah et al., 2013 [47]	AMSTAR: 11/11 Data in study: GRADE: low- to moderate-quality evidence	Systematic review and meta-narrative of RCTs, RTCs of anti-Schistosoma drugs	Trials conducted in Africa (*n* = 36), South America (*n* = 15; all in Brazil) and the Middle East (*n* = 1). 52 trials enrolling 10,269 participants in endemic areas	Intervention: praziquantel 40 mg/kg, oxamniquine 40 mg/kg	Praziquantel (single dose 40 mg/kg) vs. placebo: reduced parasitological treatment failure at 1 month (69/100; RR = 3.13, 2 trials, 414 participants). Praziquantel (single dose 30 mg/kg): RR = 1.52, 3 trials, 521 participants. Higher doses: no significant difference. Oxamniquine (single dose 40 mg/kg) vs. Placebo: reduced parasitological treatment failure at 3 months in 2 trials (68/100; RR = 8.74).
Pérez del Villar et al., 2012 [49]	AMSTAR: 11/11 Data in study: not reported. GRADE: Moderate-quality evidence	Quantitative systematic review and meta-analysis	Healthy villagers who live in areas in Africa endemic for *Sc. haematobium* and *Sc. mansoni* and in China for *Sc. Japonicum*	Intervention: prophylactic effect of artesunate or artemether vs. placebo against *Sc. haematobium*, *Sc. mansoni* and *Sc. japonica* infections. Outcomes: parasitological cure rate at 3–8 weeks; infection rate at 3–4 weeks after treatment.	Artesunate treatment (single dose: significantly lower cure rates than with praziquantel. Combined therapy of artesunate plus sulfadoxine-pyrimethamine: significantly less effective than praziquantel treatment Combination of artemisinin derivatives and praziquantel: higher cure rate than praziquantel monotherapy Artesunate or artemether: significantly better than a placebo.
Treatment efficacy of drugs for strongyloidiasis					
Henriquez-Camacho et al., 2016 [52]	AMSTAR: 11/11 GRADE: Moderate-quality evidence	Systematic review of RCTs, controlled or uncontrolled interventional studies.	Individuals with chronic infections of *St. stercoralis*; Immuno-competent patients. All ages	Intervention: ivermectin (single/double dose) vs. albendazole or thiabendazole. Outcome: elimination of infection; parasitological cure (>2 negative stool samples, 5 weeks).	Ivermectin (single/double dose) vs. albendazole: parasitological cure was higher with ivermectin, 84/100 vs. 48/100 ivermectin (RR = 1.79). Ivermectin vs. thiabendazole: little or no difference in parasitological cure, 74/100 vs. 68/100), but adverse events were less common with ivermectin (RR = 0.31) than albendazole. No serious adverse events or death reported

AMSTAR: a tool for assessing the methodological quality of systematic reviews; GRADE: Grading of Recommendations, Assessment, Development and Evaluation; LILACS: Latin American Literature in Health Sciences; RCT: randomized clinical trial; RR: Relative Risk.

**Table 3:** Characteristics of included studies on cost-effectiveness of screening and treatment of schistosomiasis and strongyloidiasis, 1993–2016.

Study	Quality	Design	Population	Intervention/Outcomes	Results
Libman et al., 1993 [70]	NA	Retrospective-cross-sectional study with cost analysis	Cohort of individuals returning from the tropics and screened in a Canadian clinic 1981–1987 Costs in 1988 CAD	Stool examination + eosinophil count + serological studies for filariasis and schistosomiasis (gold standard); vs. stool examination + eosinophil count; vs. stool examination alone; vs. stool examination + serological studies; vs. eosinophil counts only Outcome: difference in cost or resource use/cost effectiveness	Difference in resource use/costs: high-/low-prevalence locations Costs per case of schistosomiasis and/or strongyloidiasis diagnosed for each strategy: (i) CAN$4674 [€3693]; (ii) CAN$6111 [€4829]; (iii) CAN$4788 [€3783]; (iv) CAN$3737 [€2953]; (v) CAN$3307 [€2613] Cost-effectiveness (ICER or INB): no ICER calculated. Study did not include a decision analytic model
Muennig et al., 1999 [66]	NA	Decision analytic model	Large immigrant populations in which *St. stercoralis* is not endemic (one third of the sample population was from the state of New York) Costs in 1997 USD	No preventive intervention (watchful waiting) vs. universal screening vs. presumptive treatment with albendazole Outcome: difference in cost or resource use/cost effectiveness (ICER or INB) per DALY averted	Difference in resource use/costs: gross costs: USD 11,086,181 [€7,228,785] for no intervention, USD 7,290,624 [€40,203,726] per year for treatment with albendazole, USD 40,547,651 [€40,203,726] for universal screening Cost-effectiveness (ICER or INB): treatment with albendazole was cost saving compared with no intervention, universal screening had ICER of USD 159,236/DALY [€157,885/DALY] averted
Muennig et al., 2004 [67]	NA	Decision analytic model (Markov)	California and New York, two states with large immigrant populations in which *St. stercoralis* is not endemic Costs in 2000 USD	Intervention: no intervention (watchful waiting) vs. 3 or 5 days of albendazole vs. eosinophil screening vs. ivermectin Outcome: difference in cost or resource use/cost effectiveness (ICER or INB)	Difference in resource use/costs: costs per person: no intervention USD 1666 [€1611], albendazole 3 days USD 1674 [€1618], albendazole 5 days USD 1680 [€1624], screening USD 1684 [€1628], ivermectin USD 1688 [€1632] Cost-effectiveness (ICER or INB): ICERs varied based on prevalence: albendazole USD 155–1584/QALY gained [€150–1531], albendazole 5 days USD 314–3175/QALY gained [€304-3069], ivermectin USD 848–8514/QALY gained [€820-8231]. Eosinophil was documented among all prevalence groups
King et al., 2011 [65]	AMSTAR	Systematic review of efficacy of schistosomiasis treatment with praziquantel (by dose), with a Markov model estimating cost-effectiveness of various dosing strategies	Non-migrants in endemic setting; population-based or sub-population-based (e.g., schools) drug treatment of *Sc. haematobium* or *Sc. Mansoni*. Costs in 2002 & 2008 USD	Intervention: No treatment vs. single dose of praziquantel per annual treatment vs. double dose Outcome: difference in cost or resource use/cost effectiveness (ICER or INB)	Difference in resource use/costs: single dose lifetime cost: USD 23 [€19] per person; double dose: USD 46 [€35] per person. Cost-effectiveness (ICER or INB): single dose: ICER of USD 48 [€39] and USD 46 [€37] per QALY gained *for Sc. mansoni* and *Sc. haematobium*, respectively, compared with no treatment; double dose: ICERs of USD 291 [€236] and USD 433 [€351] per QALY gained respectively compared with single dose
Worrell et al., 2015 [69]	NA	Cost analysis study	Cohort of children in Kenya assessed 2010–2011. Non-migrant settings. Costs in 2010 USD	Intervention: single KK (stool examination) vs. triplicate KK vs. POC CCA (urine dipstick) Outcome: difference in cost or resource use/cost effectiveness (ICER or INB)	Difference in resource use/costs: total costs per test: single KK USD 6.89 [€5], triplicate KK USD 17.54 [€14], POC CCA USD 7.26 [€6] Cost-effectiveness (ICER or INB): no ICER calculated, this was not a decision analysis study.
Maskery et al., 2016 [68]	NA	Cost analysis study; Markov model: discount rate of 3% over 60-year time horizon; costs in 2013 USD	Average annual cohort of 27,700 Asian refugees based on Department of Homeland Security data for 2002–2011, primarily from south-east Asia and the Middle East	Intervention: no screening or treatment vs. overseas albendazole and ivermectin treatment vs. domestic screening and treatment vs. overseas albendazole and domestic screening for strongyloidiasis. Outcome: difference in cost or resource use/cost effectiveness (ICER or INB)	Difference in resource use/costs, total costs per migrant (strongyloidiasis.): no treatment USD 5.99 [€5], overseas albendazole and ivermectin USD 15.12 [€12], domestic screening and treatment USD 138.36 [€108], overseas albendazole and domestic screening for Strongyloides infection USD 78.79 [€61]. Cost-effectiveness: ICERs per QALY gained: USD 2219 for “overseas albendazole and ivermectin”, USD 32,706 [€25,422] for domestic screening and treatment, USD 18,167 [€14,121] for overseas albendazole followed by domestic screening for strongyloidiasis. All vs. no screening or treatment [€1723]

AMSTAR: A measurement tool to assess systematic reviews; CAD: Canadian dollars; CCA: circulatory cathodic antigen; GRADE: Grading of Recommendations, Assessment, Development and Evaluation; ICER: incremental cost-effectiveness ratio, INB: incremental net benefit; NA: Not Applicable KK: Kato–Katz; POC: point-of-care; USD: United States dollars.

**Table 4:** GRADE summary of findings on diagnostic tools for screening schistosomiasis, 1993–2017.

Index Test at Median Test Prevalence in Study *	Sensitivity (95% CI)	Specificity (95% CI)	Post-Test Probability of a Positive Result (95% CI)	Post-Test Probability of a Negative Result (95% CI)	Number of Studies/ Participants	Certainty of Evidence (GRADE)	Reference Standard
PCR assay (filtered urine) at 89% prevalence—*Sc. mansoni* [55]	1.00 (0.95–1.00)	1.00 (0.69–1.00)	100% (96–100)	0% (37–0)	1/89	Very Low ^a,b,c^	KK test—duplicate smears
Urine POC CCA test at 36% prevalence—*Sc. mansoni* [44]	0.89 (0.86–0.92)	0.55 (0.46–0. 65)	53% (47–60)	10% (15–7)	15/6091	Very Low ^a,b,c^	Stool microscopy
Urine POC CCA test at 30% prevalence—*Sc. mansoni* [53]	0.90 (0.84–0.94) ^d^	0.56 (0.39–0.71) ^d^	47% (37–58)	7% (15–3)	7/4584	Moderate ^a,b^	KK test
Questionnaire screening 30% prevalence—*Sc. haematobium* [50]	0.85 (0.84–0.86) ^d^	0.94 (0.94–0.94) ^d^	86% (86–86)	6% (7–6)	12/41,412	Low ^c,e^	Urine filtration/microscopy
ELISA-DRG (commercial kit) at 26% prevalence—All cases [54]	0.78 (0.61–0.90)	0.88 (0.80–0.94)	85% (65–95)	7% (13–4)	1/37	Very Low ^c,e,f^	Stool/urine microscopy
Urine heme dipstick at 27% prevalence—*Sc. haematobium* [45]	0.81 (0.73–0.83) ^d^	0.89 (0.87–0.92) ^d^	73% (67–79)	7% (10–6)	98/126,119	Low ^a,f,g^	Urine microscopy
ELISA at 24% prevalence—*Sc. japonicum* [46]	0.85 (0.83–0.87)	0.50 (0.49–0.52)	35% (34–36)	9% (10–7)	10/9014	Low ^a,f,g^	KK and Miracidium hatching test
IHA at 12% prevalence—*Sc. japonicum* [46]	0.76 (0.72–0.74) ^d^	0.73 (0.72–0.74) ^d^	28% (26–28)	4% (5–5)	15/23,411	Low ^a,b^	KK and Miracidium hatching test
ICT IgG-IgM test at 17% prevalence *Sc. mansoni* and *Sc. haematobium* [61]	0.96 (0. 91–0.99)	0.83 (0.77–0.87)	13% (9–16)	0% (0–0)	1/373	Low ^b,c^	Stool/urine microscopy/composite standard.

Population: patients with schistosomiasis or stored sera; Settings: high-/low-endemic settings; Target condition: Schistosoma spp. Infections. GRADE: Grading of Recommendations, Assessment, Development and Evaluation. Tests—CCA: circulating cathodic antigen; CI: confidence interval; DRG: DRG Instruments, Marburg, Germany; ELISA: enzyme-linked immunosorbent assay; IHA: indirect haemagglutination; KK: Kato–Katz; POC: point-of-care. * Post-test probability of test was calculated at median test prevalence obtained from individual studies. ^a^ Heterogeneity across similar studies because of several factors; downgraded because of serious inconsistency. ^b^ Use of intermediate or surrogate outcomes rather than health outcomes, hence a source of serious indirectness. ^c^ Single study design, not a randomised control trial. ^d^ Sensitivity and specificity values obtained from multiple-field study. ^e^ Use of indirect comparisons; sample population not migrants, another source of indirectness. ^f^ Very low-quality of evidence (downgraded by 1) because of serious indirectness. ^g^ Studies were insufficient to provide summary estimates for CAA tests.

**Table 5:** Accuracy of diagnostic tools for schistosomiasis at different pre-test prevalence levels, January 2010–February 2017.

Index Test	True Positives Pre-Test Probability *			False Positives Pre-Test Probability *			True Negative Pre-Test Probability *			False Negative Pre-Test Probability			% Infected Correctly Diagnosed
Test % Prevalence ^a^	2.5%	10%	30%	2.5%	10%	30%	2.5%	10%	30%	2.5%	10%	30%	
PCR assay (filtered urine)—*Sc. mansoni* [55]	25	100	300	0	0	0	975	900	700	0	0	0	100%
ICT IgG-IgM test—*Sc. haematobium*/*Sc. mansoni* [61]	24	96	288	166	153	119	809	747	581	1	4	12	96%
Urine POC CCA test—*Sc. mansoni* [53]	23	90	270	429	396	308	546	504	392	2	10	30	90%
Questionnaire screening—*Sc. haematobium* [50]	21	85	255	58	54	42	917	846	658	4	15	45	85%
ELISA-DRG (commercial kit)—*Sc. haematobuim*/*Sc. mansoni* [54]	20	78	235	47	43	34	928	857	666	5	22	65	78.3%
Urine heme dipstick—*Sc. haematobium* infections [45]	20	81	243	107	99	77	868	801	623	5	19	57	81.0%
ELISA—*Sc. japonicum* [46]	21	85	255	484	446	347	491	454	353	4	15	45	84.9%
IHA—*Sc. japonicum* [46]	19	76	227	263	243	189	712	657	511	6	24	73	75.6%

^a^ Different pre-test prevalence or probability of having schistosomiasis in an at-risk population. * Data reported as effect per 1000 migrants tested. Tests: DRG: DRG Instruments, Marburg, Germany; ELISA: enzyme-linked immunosorbent assay; ICT: Immuno chromatographic test; IHA: Indirect haemagglutination; PCR: Polymerase chain reaction assay; POC: Point of care.

**Table 6:** GRADE summary of findings on diagnostic tools for screening strongyloidiasis, January 1993–February 2017.

Index Test—at 10% Prevalence *	Sensitivity (95% CI)	Specificity (95% CI)	Post-Test Probability of a Positive Result (95% CI)	Post-Test Probability of a Negative Result (95% CI)	Number of Studies/ Participants	Certainty of Evidence (GRADE)	Reference Standard
Baermann method [51]	0.72 (0.67–0.76) ^a^	1.00 (1.00–1.00) ^a^	100% (100–100)	3% (4–3)	9/2459	Moderate ^b,c^	Combination of diagnostic tests
Agar plate—10% prevalence [51]	0.89 (0.86–0.92) ^a^	1.00 (1.00–1.00) ^a^	100% (100–100)	1% (2–1)	10/3563	Moderate ^b,c^	Combination of diagnostic tests
NIE LIPS [62] ^d^	0.85 (0.79–0.92)	0.95 (0.93–0.98)	65% (56–84)	2% (2–1)	1/399	Low ^e,f,g^	Stool microscopy or culture
IVD ELISA—commercial test [62]	0.92 (0.87–0.97)	0.97 (0.96–0.99)	77% (71–92)	1% (1–0)	1/399	Low ^e,f,h^	Stool microscopy
IFAT [62]	0.94 (0.90–0.98)	0.87 (0.83–0.91)	45% (37–55)	1% (1–0)	1/399	Low ^e,f,h^	Stool microscopy and culture
Bordier-ELISA—commercial kit [62]	0.91 (0.86–0.96)	0.94 (0.91–0.96)	63% (52–77)	1% (2–0)	1/193	Low ^e,f,h^	Kato–Katz, Flotac, and Baermann method
SS-NIE-1 ELISA [63]	0.95 (0.92–0.97)	0.93 (0.90–0.96)	60% (71–73%)	1% (1–0)	1/583	Low ^f,g,i^	Stool microscopy and culture

Notes: Population: patients with strongyloidiasis or sera infected with *St. stercoralis*; Settings: low-/high-endemic areas; Target condition: strongyloidiasis (test prevalence 10%). Cost effectiveness: serological testing may be cost-effective relative to stool and eosinophil testing for both strongyloidiasis and schistosomiasis, because of superior test performance characteristics. Tests: ELISA: enzyme-linked immunosorbent assay; GRADE: Grading of Recommendations, Assessment, Development and Evaluation; IFAT: indirect fluorescent antibody technique; IVD: Invitro diagnostic test; LIPS: luciferase immunoprecipitation system; NIE: a 31-kDa recombinant antigen from *St. stercoralis*. * Post-test probability of test was calculated at 10% prevalence for all the tests. ^a^ Sensitivity and specificity values obtained from a multiple-field study. ^b^ Evidence was downgraded because of serious inconsistencies and heterogeneity. ^c^ Heterogeneity between studies; use of intermediate or surrogate outcomes rather than health outcomes. ^d^ Test result with a primary standard. ^e^ Absence of a reliable gold standard for diagnosis of *S. stercoralis* infection. The review did not describe the specific gold standard used in the included studies for each test. ^f^ Single study design. ^g^ Samples were classified according to a composite reference standard, a procedure suggested for evaluation of diagnostic tests when there is no gold standard. ^h^ Use of intermediate or surrogate outcomes rather than health outcomes. ^i^ The inter-assay coefficient of variation was determined to be 22% for the low-positive control serum and 10% for the medium-positive control serum.

**Table 7:** Accuracy of diagnostic tools for strongyloidiasis at different pre-test prevalence levels, 2012–February 2017.

Index tests	True-Positives Pre-Test Probability ^a^			False-Positives Pre-Test Probability ^a^			True-Negatives Pre-Test Probability ^a^			False-Negatives Pre-Test Probability ^a^			% Infected Correctly Diagnosed
Test % Prevalence ^b^	2.5%	10%	30%	2.5%	10%	30%	2.5%	10%	30%	2.5%	10%	30%	
Baermann method [51]	18	72	216	0	0	0	975	900	700	7	28	84	72%
Agar plate [51]	22	89	267	0	0	0	975	900	700	3	11	33	89%
NIE-LIPS [62]	21	85	255	49	45	35	926	855	665	4	15	45	85.1%
IVD-ELISA (commercial test) [62]	23	92	276	29	27	21	946	873	679	2	8	24	92%
IFAT [62]	23	94	282	127	117	91	848	783	609	2	6	18	93.8%
Bordier-ELISA (commercial kit) [62]	23	91	272	58	54	42	917	846	658	2	9	28	90.7%
SS-NIE-1 ELISA [63]	24	95	285	68	63	49	907	837	651	1	5	15	95%

ELISA: enzyme-linked immunosorbent assay; IFAT: indirect fluorescent antibody technique; IVD: Invitro diagnostic test; LIPS: luciferase immunoprecipitation system; NIE: 31-kDa recombinant antigen from *St. stercoralis.* ^a^ Data reported as effect per 1000 migrants tested. ^b^ pre-test prevalence or probability of having schistosomiasis in an at-risk population.

**Table 8:** GRADE summary of findings of different schistosomiasis treatments vs. placebo, 2010–2016.

Outcomes	Anticipated Absolute Effects ^a^ (95% CI)		Relative Chance of Cure (95% CI)	Number of Participants/Studies	Certainty of the Evidence (GRADE)
	Risk with Placebo per 1000	Cure with Intervention Drug			
Parasitological failure at 1 to 2 months (praziquantel 40 mg/kg single dose) [48]	908	381 (263–562)	RR 0.42 (0.29 to 0.58)	864/7 RCTs	High
Parasitological cure at 1 month ^b^—*Sc. mansoni* infections (praziquantel 40 mg/kg single dose) [47]	337	1000 (347–1000)	RR 3.13 (1.03–9.53)	414/2 RCTs	Moderate ^c^
Microhaematuria at 8 weeks (praziquantel 40 mg/kg single dose) [48]	281	149 (93–236)	RR 0.53 (0.33–0.84)	119/1 RCT	Low ^d,e,f^
Infection rate of *Sc. japonicum* (artemether monotherapy 6 mg/kg) [49]	175	44 (28–70)	RR 0.25 (0.16–0.40)	8051/13 RCTs	Moderate ^c^
Parasitological cure rate of *Schistosoma species*. (Artesunate—monotherapy (4 mg/kg daily for three consecutive days)) [49]	615 *	302 (172–459)	RR 0.49 (0.28–0.75)	800/7 RCTS	Moderate ^c^
Adverse events, minor (praziquantel 40 mg/kg single dose) [49]	None	None	Not estimable	1591/9 RCTs	Low ^d^

CI: confidence interval; GRADE: Grading of Recommendations, Assessment, Development and Evaluation; RR: risk ratio; RTC: randomized controlled trial. * praziquantel 40 mg/kg once. ^a^ The risk in the intervention group per 1000 persons treated (95% CI) was based on the assumed risk in the comparison group and the relative effect of the intervention (and its 95% CI). ^b^ Treatment of only *Sc. mansoni* infections reported. ^c^ Downgraded by 1 for indirectness: only two trials from limited settings evaluated this comparison. ^d^ The trial was under-powered; downgraded by 1. ^e^ Only a single trial reported this outcome. ^f^ Publication bias was unclear.

**Table 9:** GRADE summary of findings on ivermectin (200 mg/kg) vs. albendazole or thiabendazole for the treatment of strongyloidiasis, and certainty of evidence on treatment efficacy, benefits and harms, 2010–2016.

Outcomes	Anticipated Absolute Effects (95% CI)		Relative Chance of Cure (95% CI) ^b^	Number of Participants/Studies	Certainty of the Evidence (GRADE)
	Cure with Comparator Drug per 1000 ^a^	Cure with Intervention Drug—Ivermectin (200 mg/kg) ^b^			
Cure overall assessed at 5 weeks—albendazole [52]	480	840 (720–980)	RR 1.79 (1.55–2.08)	478/4 RCTs	Moderate ^d^
Adverse events assessed at 5 weeks—albendazole [52]	260	210 (150–290)	RR 0.80 (0.59–1.09)	518/4 RCTs	Low ^c,g^
Cure overall assessed at 11 weeks—thiabendazole [52]	690	740 (660–820)	RR 1.07 (0.96–1.20)	467/3 RCTs	Moderate ^e^
Adverse events assessed at 11 weeks—thiabendazole [52]	730	230 (150–360)	RR 0.31 (0.20–0.50)	507/3 RCTs	Moderate ^f^

PICO—Patient or population: sersons with Strongyloides stercoralis infection; Setting: south-east-Asia, America and Europe; Intervention: ivermectin; Comparison: albendazole and thiabendazole. CI: confidence interval; GRADE: Grading of Recommendations, Assessment, Development and Evaluation; RR: risk ratio; RTC: randomized controlled trial. ^a^ Albendazole or thiabendazole. ^b^ The risk in the intervention group per 1000 persons treated (95% CI) was based on the assumed risk in the comparison group and the relative effect of the intervention (and its 95% CI). ^c^ No method of allocation concealment in two trials and no method of allocation described. ^d^ Two trials did not conceal allocation and no method of allocation was described. ^e^ Two trials did not conceal allocation and no method of allocation was described in one trial. ^f^ Two trials did not conceal allocation and no method of allocation was described. ^g^ Wide range of estimates in three trials could include substantive fewer events.

**Figure A1:**
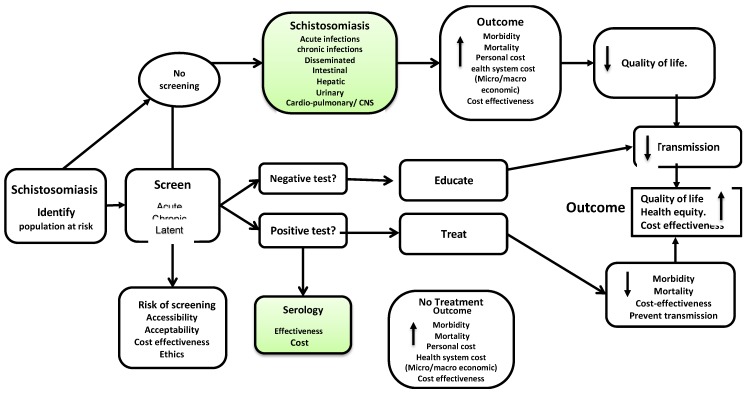
Analytic framework for screening and treatment of schistosomiasis in migrants.

**Figure A2:**
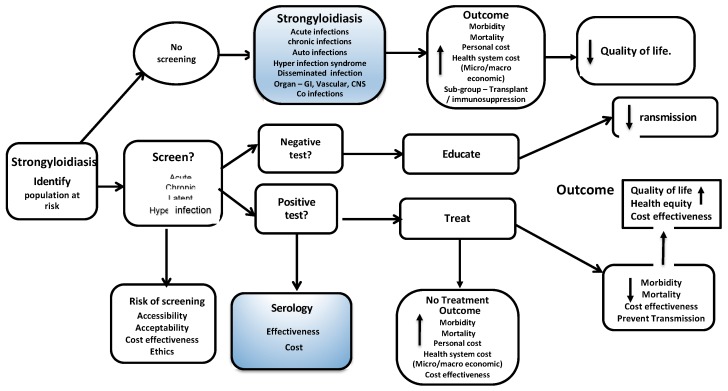
Analytic framework for screening and treatment of strongyloidiasis in migrants.

## Appendix B. List of Sites and Literature Search Strategy

**1. Literature search strategy for systematic review**

The used search strategies for the identification of systematic reviews are listed here.

A. Database: Ovid MEDLINE(R) 1946 to Present with Daily Update

Search Date: 15 April 2016

--------------------------------------------------------------------------------

1. exp Schistosoma/ (15595)
2. bilharzia$.tw. (2431)
3. exp Schistosomiasis/ (21432)
4. schistosom$.tw. (25367)
5. katayama fever$.tw. (30)
6. or/1–5 (30014)
7. Strongyloides/ (985)
8. Strongyloides stercoralis/ (1044)
9. Strongyloidiasis/ (3301)
10. strongyloid$.tw. (3988)
11. or/7–10 (4959)
12. 6 or 11 (34621)
13. exp Mass Screening/ (107821)
14. (screened or screening?) tw. (417896)
15. Early Diagnosis/ (19041)
16. (detected or detection? or diagnos$ or discover$ or indentif$) tw. (2972048)
17. exp Population Surveillance/ (56090)
18. (disease? adj2 surveillance) tw. (4053)
19. Contact Tracing/ (3521)
20. contact tracing tw. (1152)
21. or/13–20 (3301561)
22. meta analysis mp, pt. (91365)
23. review pt. (2035657)
24. search$ tw. (253765)
25. or/22–24 (2222329)
26. animals/ not (humans/ and animals/) (4194238)
27. 25 not 26 (2065589)
28. 12 and 21 and 27 (711)
29. 28 and (2010$ or 2011$ or 2012$ or 2013$ or 2014$ or 2015$ or 2016$) ed. (222)
30. remove duplicates from 29 (218)

***************************

B. Database: Embase <1980 to 2016 April 14>

Search Date: 15 April 2016

--------------------------------------------------------------------------------

1. exp Schistosoma/ (19846)
2. bilharzia$.tw. (2115)
3. exp schistosomiasis/ (20241)
4. schistosom$.tw. (26744)
5. katayama fever$.tw. (40)
6. or/1–5 (33204)
7. Strongyloides/ (1220)
8. Strongyloides stercoralis/ (2315)
9. strongyloidiasis/ (3835)
10. strongyloid$.tw. (4704)
11. or/7–10 (6600)
12. 6 or 11 (39071)
13. exp mass screening/ (178654)
14. (screened or screening?).tw. (614882)
15. early diagnosis/ (82347)
16. parasite identification/ (13161)
17. ((case? or early or parasit$) adj5 (detected or detection? or diagnos$ or discover$ or egg or indentif$)).tw. (385884)
18. exp health survey/ (182738)
19. (disease? adj2 surveillance).tw. (5156)
20. contact examination/ (2830)
21. contact tracing.tw. (1448)
22. or/13–21 (1237076)
23. meta analys$.mp. (167508)
24. search$.tw. (362044)
25. review.pt. (2131214)
26. or/23–25 (2472677)
27. (exp animal/ or animal.hw. or nonhuman/) not (exp human/ or human cell/ or (human or humans) ti.) (5499319)
28. 26 not 27 (2251777)
29. 12 and 22 and 28 (455)
30. 29 and (2010$ or 2011$ or 2012$ or 2013$ or 2014$ or 2015$ or 2016$) dd. (195)
31. remove duplicates from 30 (190)

***************************

C. Database: EBSCO CINAHL <1970 to April 2016>

Search Date: 15 April 2016

--------------------------------------------------------------------------------

# Query Limiters/Expanders Last Run Via Results

S28 S24 AND S27 129

S27 S25 OR S26 2,596,403

S26 EM 2010 or EM 2011 or EM 2012 or EM 2013 or EM 2014 or EM 2015 or EM 2016 2,415,478

S25 PY 2010 or PY 2011 or PY 2012 or PY 2013 or PY 2014 or PY 2015 or PY 2016 2,346,296

S24 S9 AND S17 AND S23 253

S23 S18 OR S19 OR S20 OR S21 OR S22 221,252

S22 (TI meta analy * or AB meta analy *) 29,697

S21 (MH “Meta-Analysis”) 24,939

S20 PT review 141,448

S19 PT systematic review 53,358

S18 (MH “Systematic Review”) 37,435

S17 S10 OR S11 OR S12 OR S13 OR S14 OR S15 OR S16 1,246,183

S16 TX contact tracing 2230

S15 TX (disease * or population) N2 surveillance 23,893

S14 (MH “Population Surveillance+”) 5949

S13 TX (detected or detection * or diagnos * or discover * or indentif *) 1,184,038

S12 (MH “Early Diagnosis”) 4472

S11 TI ((screened or screening *) OR AB (screened or screening *)) 78,236

S10 (MH “Health Screening+”) 62,744

S9 S5 OR S8 5460

S8 S6 OR S7 4460

S7 TX strongyloid * 529

S6 (MH “Helminthiasis+”) 4132

S5 S1 OR S2 OR S3 OR S4 1931

S4 TX katayama fever 25

S3 TX bilharzia * 175

S2 TX schistosome * 1871

S1 (MH “Schistosomiasis+”) 756

***************************

D. Databases: Database of Abstracts of Reviews of Effects (DARE) and Cochrane Database of Systematic Reviews (CDSR)

Search Date: 15 April 2016

--------------------------------------------------------------------------------

ID Search

#1 MeSH descriptor: [Schistosoma] explode all trees

#2 bilharzia *

#3 MeSH descriptor: [Schistosomiasis] explode all trees

#4 schistosom *

#5 katayama fever

#6 #1 or #2 or #3 or #4 or #5

#7 MeSH descriptor: [Strongyloides] this term only

#8 MeSH descriptor: [Strongyloides stercoralis] this term only

#9 MeSH descriptor: [Strongyloidiasis] this term only

#10 strongyloid *

#11 #7 or #8 or #9 or #10

#12 #6 or #11

#13 #12 in Other Reviews

#14 #12 in Cochrane Reviews (Reviews and Protocols)

***************************

**2. Literature search strategy for systematic search for cost-effectiveness studies**

The used search strategies for the identification of systematic reviews on cost-effectiveness are listed here.

A. Database: Ovid MEDLINE(R) Epub Ahead of Print <May Week 3 2016>, Ovid MEDLINE(R) 1946 to Present with Daily Update

Search Date: 31 May 2016

--------------------------------------------------------------------------------

1. exp Schistosoma/ (15714)
2. bilharzia$.tw. (2438)
3. exp Schistosomiasis/ (21583)
4. schistosom$.tw. (25722)
5. katayama fever$.tw. (30)
6. or/1–5 (30381)
7. Strongyloides/ (990)
8. Strongyloides stercoralis/ (1056)
9. Strongyloidiasis/ (3319)
10. strongyloid$.tw. (4079)
11. or/7–10 (5051)
12. 6 or 11 (35067)
13. exp Mass Screening/ (108535)
14. (screened or screening? or tested or testing or tests).tw. (1734474)
15. Early Diagnosis/ (19350)
16. (detected or detection? or diagnos$ or discover$ or indentif$).tw. (3053822)
17. exp Population Surveillance/ (56687)
18. (disease? adj2 surveillance).tw. (4195)
19. Contact Tracing/ (3563)
20. contact tracing.tw. (1176)
21. or/13–20 (4387118)
22. meta analysis.mp,pt. (96759)
23. review.pt. (2060867)
24. search$.tw. (266775)
25. guideline.pt. (15780)
26. guideline/ (15780)
27. guidelines as topic/ (34071)
28. practice guideline.pt. (21216)
29. practice guideline/ (21216)
30. practice guidelines as topic/ (91792)
31. (CPG or CPGs or guidance or guideline? or recommend$ or standard?).ti. (147179)
32. exp clinical pathway/ (5273)
33. exp clinical protocol/ (139345)
34. ((care or clinical) adj2 pathway?).tw. (5129)
35. or/22–34 (2572065)
36. 12 and 21 and 35 (960)
37. animals/ not (humans/ and animals/) (4215704)
38. 36 not 37 (838)
39. 38 and (2010$ or 2011$ or 2012$ or 2013$ or 2014$ or 2015$ or 2016$).ed. (271)
40. remove duplicates from 39 [reviews and guidelines] (261)
41. exp "costs and cost analysis"/ (197942)
42. cost$.mp. (467877)
43. cost effective$.tw. (83090)
44. cost benefit analys$.mp. (67319)
45. health care costs.mp. (37157)
46. or/41–45 (477217)
47. 12 and 21 and 46 (260)
48. animals/ not (humans/ and animals/) (4215704)
49. 47 not 48 (222)
50. 49 and (2010$ or 2011$ or 2012$ or 2013$ or 2014$ or 2015$ or 2016$).ed. (82)
51. remove duplicates from 50 (78)

***************************

B. Database: Embase <1974 to 2016 Week 22>

Search Date: 31 May 2016

--------------------------------------------------------------------------------

1. exp Schistosoma/ (21727)
2. bilharzia$.tw. (2492)
3. exp schistosomiasis/ (21930)
4. schistosom$.tw. (29047)
5. katayama fever$.tw. (42)
6. or/1–5 (36157)
7. Strongyloides/ (1229)
8. Strongyloides stercoralis/ (2447)
9. strongyloidiasis/ (3986)
10. strongyloid$.tw. (4977)
11. or/7–10 (6962)
12. 6 or 11 (42352)
13. exp mass screening/ (182895)
14. (screened or screening? or tested or testing or tests).tw. (2429856)
15. early diagnosis/ (83110)
16. parasite identification/ (13222)
17. ((case? or early or parasit$) adj5 (detected or detection? or diagnos$ or discover$ or egg or indentif$)).tw. (405389)
18. exp health survey/ (184236)
19. (disease? adj2 surveillance).tw. (5253)
20. contact examination/ (2867)
21. contact tracing.tw. (1512)
22. or/13-21 (2999272)
23. meta analys$.mp. (170914)
24. search$.tw. (371898)
25. review.pt. (2163187)
26. guideline.pt. (0)
27. guideline/ (144)
28. guidelines as topic/ (229895)
29. practice guideline.pt. (0)
30. practice guideline/ (275502)
31. practice guidelines as topic/ (171091)
32. (CPG or CPGs or guidance or guideline? or recommend$ or standard?).ti. (203285)
33. exp clinical pathway/ (6983)
34. exp clinical protocol/ (75932)
35. ((care or clinical) adj2 pathway?).tw. (9455)
36. or/23–35 (2900847)
37. 12 and 22 and 36 (824)
38. (exp animal/ or animal.hw. or nonhuman/) not (exp human/ or human cell/ or (human or humans).ti.) (5865460)
39. 37 not 38 (678)
40. 39 and (2010$ or 2011$ or 2012$ or 2013$ or 2014$ or 2015$ or 2016$).dd. (304)
41. remove duplicates from 40 [reviews and guidelines] (295)
42. cost effectiveness analysis/ (114264)
43. cost.tw. (387431)
44. costs.tw. (208732)
45. or/42–44 (544771)
46. 12 and 22 and 45 (274)
47. (exp animal/ or animal.hw. or nonhuman/) not (exp human/ or human cell/ or (human or humans).ti.) (5865460)
48. 46 not 47 (223)
49. 48 and (2010$ or 2011$ or 2012$ or 2013$ or 2014$ or 2015$ or 2016$).dd. (115)
50. remove duplicates from 49 [costing] (111)

***************************

C. Databases: Database of Abstracts of Reviews of Effects (DARE) and Cochrane Database of Systematic Reviews (CDSR) and NHS EED

Search Date: 31 May 2016

--------------------------------------------------------------------------------

ID Search

#1 MeSH descriptor: [Schistosoma] explode all trees

#2 bilharzia*

#3 MeSH descriptor: [Schistosomiasis] explode all trees

#4 schistosom*

#5 katayama fever

#6 #1 or #2 or #3 or #4 or #5

#7 MeSH descriptor: [Strongyloides] this term only

#8 MeSH descriptor: [Strongyloides stercoralis] this term only

#9 MeSH descriptor: [Strongyloidiasis] this term only

#10 strongyloid*

#11 #7 or #8 or #9 or #10

#12 #6 or #11

#13 #12 in Other Reviews

#14 #12 in Cochrane Reviews (Reviews and Protocols)

#15 #12 in Economic Evaluations

***************************

D. Database: EBSCO CINAHL <1970 to May 2016>

Search Date: 31 May 2016

--------------------------------------------------------------------------------

# Query Limiters/Expanders Last Run Via Results

S38 S32 AND S37 38

S37 S9 AND S17 AND S36 76

S36 S34 OR S35 139,767

S35 TI (cost OR costs) OR AB (cost OR costs) 89,616

S34 (MH “Costs and Cost Analysis+”) 82,915

S33 S29 AND S32 164

S32 S30 OR S31 2,653,954

S31 EM 2010 or EM 2011 or EM 2012 or EM 2013 or EM 2014 or EM 2015 or EM 2016 2,445,432

S30 PY 2010 or PY 2011 or PY 2012 or PY 2013 or PY 2014 or PY 2015 or PY 2016 2,403,611

S29 S9 AND S17 AND S28 307

S28 S18 OR S19 OR S20 OR S21 OR S22 OR S23 OR S24 OR S25 OR S26 OR S27 348,353

S27 TX (care or clinical) N2 pathway * 15,555

S26 TI (CPG or CPGs or guidance or guideline * or recommend * or standard *) 79,261

S25 (MH “Critical Path”) 4120

S24 PT Practice Guidelines 9487

S23 (MH “Practice Guidelines”) 53,690

S22 (TI meta analy * or AB meta analy *) 30,542

S21 (MH “Meta Analysis”) 25,200

S20 PT review 144,019

S19 PT systematic review 53,350

S18 (MH “Systematic Review”) 37,846

S17 S10 OR S11 OR S12 OR S13 OR S14 OR S15 OR S16 1,801,344

S16 TX contact tracing 2236

S15 TX (disease * or population) N2 surveillance 24,089

S14 (MH “Population Surveillance+”) 6026

S13 TX (detected or detection * or diagnos * or discover * or indentif *) 1,195,388

S12 (MH “Early Diagnosis”) 4553

S11 TX (screened or screening * or tested or testing or tests) 1,102,848

S10 (MH “Health Screening+”) 63,147

S9 S5 OR S8 5501

S8 S6 OR S7 4500

S7 TX strongyloid * 537

S6 (MH “Helminthiasis+”) 4167

S5 S1 OR S2 OR S3 OR S4 1942

S4 TX katayama fever 24

S3 TX bilharzia * 175

S2 TX schistosome * 1881

S1 (MH “Schistosomiasis+”) 764

***************************

E. Databases: PubMed

Search Date: 31 May 2016

--------------------------------------------------------------------------------

((((((((schistosome * or bilharzia * or katayama or strongyloid *))) AND ((screened or screening * or tested or testing or tests)))) AND (((CPG or CPGs or guidance or guideline * or metaanalysis or meta-analysis or recommend * or review or standard or standards)))) AND ((publisher [3]))))) (8)

((((((((schistosome * or bilharzia * or katayama or strongyloid *))) AND ((screened or screening * or tested or testing or tests)))) AND (((cost or costs)))) AND ((publisher [3]))))) (2)

***************************

**3. Update Literature strategy for primary studies on diagnostic or screening tools for schistosomiasis.**

A. Database: Ovid MEDLINE(R)—1946 to February 2017.

1. Schistosomiasis/ (13485)
2. Schistosomiasis.mp. (24533)
3. snail fever.mp. (10)
4. schistosome *.mp. (5528)
5. exp "Sensitivity and Specificity"/ (495027)
6. sensitivity.tw. (638974)
7. specificity.tw. (379605)
8. ((pre-test or pretest) adj probability).tw. (1695)
9. post-test probability.tw. (441)
10. predictive value$.tw. (85102)
11. likelihood ratio$.tw. (11639)
12. or/5–11 (1217873)
13. or/1–4 (26340)
14. 12 and 13 (1493)
15. limit 14 to humans (1112)
16. from 15 keep 1001–1112 (112)

A. Database: EMBASE—up to February 2017

#16 #14 AND ‘human’/de AND [embase]/lim NOT [medline]/lim 308

#15 #14 AND ‘human’/de 1489

#14 #5 AND #13 2534

#13 #6 OR #7 OR #8 OR #9 OR #10 OR #11 OR #12 1688887

#12 ‘sensitivity and sensibility’ 982

#11 ‘sensitivity’ 1132406

#10 ‘specificity’ 719846

#9 ‘pretest posttest design’ 2331

#8 ‘predictive value’ 161458

#7 ‘likelihood ratio’ 11832

#6 ‘diagnostic accuracy’ 220669

#5 #1 OR #2 OR #3 OR #4 35984

#4 ‘snail fever’ 9

#3 ‘schistosome’ 4643

#2 ‘schistosoma’ 25091

#1 ‘schistosomiasis’/exp 22890

B. Database: COCHRANE LIBRARY- up to February 2017

ID Search Hits

#1 MeSH descriptor: [Schistosomiasis] explode all trees 295

#2 Schistosomiasis 497

#3 snail fever 3

#4 schistosome * 50

#5 #1 or #2 or #3 or #4 506

#6 MeSH descriptor: [Diagnosis] explode all trees 298999

#7 diagno * 129750

#8 #6 or #7 367644

#9 #5 and #8 220

C. Database: CINAHL—up to February 2017

S12 S4 AND S11

S11 S5 OR S6 OR S7 OR S8 OR S9 OR S10

S10 likelihood ratio$

S9 predictive value$

S8 post-test probability

S7 sensitivity and specificity

S6 specificity

S5 sensitivity

S4 S1 OR S2 OR S3

S3 schistosoma

S2 schistosome *

S1 Schistosomiasis

D. Database: LILACS – up to February 2017

(tw:((tw:(esquistosomiasis)) OR (tw:(bilharziasis)) OR (tw:(schistosoma)))) AND (tw:((tw:(diagnostico)) OR (tw:(deteccion)))) AND (instance:”regional”) AND (db:(“LILACS” OR “colecionaSUS” OR “IBECS” OR “SES-SP” OR “MedCarib” OR “CUMED”) AND clinical_aspect:(“diagnosis”) AND limit:(“humans”))

***************************

**4. Update Literature strategy for primary studies on diagnostic or screening tools for strongyloidiasis**

A. Database: Ovid MEDLINE(R)—1946 to February 2017

1. Strongyloidiasis/ (3403)
2. Strongyloidiasis.mp. (3747)
3. Strongyloides stercoralis/ (1098)
4. Strongyloides stercoralis.mp. (2142)
5. or/1–4 (4376)
6. exp “Sensitivity and Specificity”/ (494358)
7. sensitivity.tw. (637846)
8. specificity.tw. (379066)
9. ((pre-test or pretest) adj probability).tw. (1689)
10. post-test probability.tw. (438)
11. predictive value$.tw. (84929)
12. likelihood ratio$.tw. (11613)
13. or/6–12 (1216076)
14. 5 and 13 (247)
15. limit 14 to humans (207)

B. Database: EMBASE—up to February 2017

No. Query Results

#14 #12 AND [embase]/lim NOT [medline]/lim AND ‘human’/de 136

#13 #12 AND [embase]/lim NOT [medline]/lim 156

#12 #3 AND #11 472

#11 #4 OR #5 OR #6 OR #7 OR #8 OR #9 OR #10 1686971

#10 ‘diagnostic accuracy’ 220414

#9 ‘likelihood ratio’ 11815

#8 ‘predictive value’ 161090

#7 ‘pretest posttest design’ 2315

#6 ‘specificity’ 719056

#5 ‘sensitivity’ 1131076

#4 ‘sensitivity and sensibility’ 981

#3 #1 OR #2 5662

#2 ‘strongyloides stercoralis’ 3193

#1 ‘strongyloidiasis’/exp 4162

C. Database: COCHRANE LIBRARY—up to February 2017

ID Search Hits

#1 MeSH descriptor: [Strongyloidiasis] explode all trees 28

#2 Strongyloidiasis 53

#3 MeSH descriptor: [Strongyloides stercoralis] explode all trees 12

#4 Strongyloides stercoralis 47

#5 #1 or #2 or #3 or #4 72

#6 MeSH descriptor: [Diagnosis] explode all trees 298999

#7 diagno * 129739

#8 #6 or #7 367633

#9 #5 and #8 38

D. Database: CINAHL—up to February 2017

Términos de la búsqueda Opciones de búsqueda

S11 (S4 OR S5 OR S6 OR S7 OR S8 OR S9) AND (S3 AND S10)

S10 S4 OR S5 OR S6 OR S7 OR S8 OR S9

S9 likelihood ratio$

S8 predictive value$

S7 post-test probability

S6 sensitivity and specificity

S5 specificity

S4 sensitivity

S3 S1 OR S2

S2 strongyloides stercoralis

S1 strongyloidiasis

E. Database: LILACS—up to February 2017

(tw:((tw:(estrongiloidiasis)) OR (tw:(Strongyloides stercoralis)))) AND (tw:((tw:(diagnostico)) OR (tw:(deteccion))))

***************************

## References

[1] Puthiyakunnon S., Boddu S., Li Y., Zhou X., Wang C., Li J., Chen X. (2014). Strongyloidiasis—An insight into its global prevalence and management. PLoS Negl. Trop. Dis..

[2] Riccardi N., Nosenzo F., Peraldo F., Sarocchi F., Taramasso L., Traverso P., Viscoli C., Di Biagio A., Derchi L.E., De Maria A. (2017). Increasing prevalence of genitourinary schistosomiasis in Europe in the Migrant Era: Neglected no more?. PLoS Negl. Trop. Dis..

[3] Murray C.J., Vos T., Lozano R., Naghavi M., Flaxman A.D., Michaud C., Ezzati M., Shibuya K., Salomon J.A., Abdalla S. (2012). Disability-adjusted life years (DALYs) for 291 diseases and injuries in 21 regions, 1990–2010: A systematic analysis for the Global Burden of Disease Study 2010. Lancet.

[4] King C.H. (2010). Parasites and poverty: The case of schistosomiasis. Acta Trop..

[5] Zoni A.C., Catalá L., Ault S.K. (2016). Schistosomiasis Prevalence and Intensity of Infection in Latin America and the Caribbean Countries, 1942–2014: A Systematic Review in the Context of a Regional Elimination Goal. PLoS Negl. Trop. Dis..

[6] Schar F., Trostdorf U., Giardina F., Khieu V., Muth S., Marti H., Vounatsou P., Odermatt P. (2013). Strongyloides stercoralis: Global Distribution and Risk Factors. PLoS Negl. Trop. Dis..

[7] Bisoffi Z., Buonfrate D., Montresor A., Requena-Mendez A., Munoz J., Krolewiecki A.J., Gotuzzo E., Mena M.A., Chiodini P.L., Anselmi M. (2013). Strongyloides stercoralis: A plea for action. PLoS Negl. Trop. Dis..

[8] Adenowo A.F., Oyinloye B.E., Ogunyinka B.I., Kappo A.P. (2015). Impact of human schistosomiasis in sub-Saharan Africa. Braz. J. Infect. Dis..

[9] Hotez P.J., Alvarado M., Basanez M.G., Bolliger I., Bourne R., Boussinesq M., Brooker S.J., Brown A.S., Buckle G., Budke C.M. (2014). The global burden of disease study 2010: Interpretation and implications for the neglected tropical diseases. PLoS Negl. Trop. Dis..

[10] Beltrame A., Buonfrate D., Gobbi F., Angheben A., Marchese V., Monteiro G.B., Bisoffi Z. (2017). The hidden epidemic of schistosomiasis in recent African immigrants and asylum seekers to Italy. Eur. J. Epidemiol..

[11] Khan K., Sears J., Chan A., Rashid M., Greenaway C., Stauffer W., Narasiah L., Pottie K. (2011). Canadian Collaboration for Immigrant and Refugee Health (CCIRH). Strongyloides and Schistosoma: Evidence review for newly arriving immigrants and refugee. The Canadian Collaboration for Immigrant and Refugee Health. Appendix 8: Intestinal Parasites.

[12] Wilson J.M.G., Jungner G., Organization W.H. (1968). Principles and Practice of Screening for Disease.

[13] Colley D.G., Bustinduy A.L., Secor W.E., King C.H. (1969). Human schistosomiasis. Lancet.

[14] Deniaud F., Rouesse C., Collignon A., Domingo A., Rigal L. (2010). Failure to offer parasitology screening to vulnerable migrants in France: Epidemiology and consequences. Sante (Montrouge, France).

[15] Ross A.G., McManus D.P., Farrar J., Hunstman R.J., Gray D.J., Li Y.S. (2012). Neuroschistosomiasis. J. Neurol..

[16] Buonfrate D., Requena-Mendez A., Angheben A., Munoz J., Gobbi F., Van Den Ende J., Bisoffi Z. (2013). Severe strongyloidiasis: A systematic review of case reports. BMC Infect. Dis..

[17] Kim J.H., Kim D.S., Yoon Y.K., Sohn J.W., Kim M.J. (2016). Donor-Derived Strongyloidiasis Infection in Solid Organ Transplant Recipients: A Review and Pooled Analysis. Transp. Proc..

[18] Berry A., Paris L., Boissier J., Caumes E. (2016). Schistosomiasis Screening of Travelers to Corsica, France. Emerg. Infect. Dis..

[19] Requena-Mendez A., Chiodini P., Bisoffi Z., Buonfrate D., Gotuzzo E., Munoz J. (2013). The laboratory diagnosis and follow up of strongyloidiasis: A systematic review. PLoS Negl. Trop. Dis..

[20] Greaves D., Coggle S., Pollard C., Aliyu S.H., Moore E.M. (2013). Strongyloides stercoralis infection. BMJ.

[21] Deniaud F., Legros P., Collignon A., Prevot M., Domingo A., Ayache B. (2008). Targeted screening proposed in 6 migrant worker housing units in Paris in 2005: Feasibility and impact study. Sante Publique.

[22] Chernet A., Kling K., Sydow V., Kuenzli E., Hatz C., Utzinger J., van Lieshout L., Marti H., Labhardt N.D., Neumayr A. (2017). Accuracy of diagnostic tests for Schistosoma mansoni infection in asymptomatic Eritrean refugees: Serology and POC-CCA against stool microscopy. Clin. Infect. Dis..

[23] Weerakoon K.G., Gobert G.N., Cai P., McManus D.P. (2015). Advances in the Diagnosis of Human Schistosomiasis. Clin. Microbiol. Rev..

[24] Agbata E.N., Padilla P.F., Agbata I.N., Armas L.H., Sola I., Pottie K., Alonso-Coello P. (2018). Migrant Healthcare Guidelines: A Systematic Quality Assessment. J. Immigr. Minor. Health.

[25] Eurostat Eurostat migr_resfirst, m.r. Residence permits statistics. https://ec.europa.eu/eurostat/documents/2995521/9333446/3-25102018-AP-EN.pdf/3fa5fa53-e076-4a5f-8bb5-a8075f639167.

[26] European Centre for Disease Prevention and Control (2017). Monitoring implementation of the Dublin Declaration on Partnership to Fight HIV/AIDS in Europe and Central Asia: 2017 Progress Report Stockholm.

[27] Eurostat Eurostat migr_asydcfsta, t. https://ec.europa.eu/eurostat/statistics-explained/pdfscache/13562.pdf.

[28] European Parliament (2017). EU Migrant Crisis: Facts and Figures. http://www.europarl.europa.eu/news/en/headlines/society/20170629STO78630/eu-migrant-crisis-facts-and-figures.

[29] Gushulak B.D., MacPherson D.W. (2004). Population mobility and health: An overview of the relationships between movement and population health. J. Travel Med..

[30] Beknazarova M., Whiley H., Ross K. (2016). Strongyloidiasis: A disease of socioeconomic disadvantage. Int. J. Environ. Res. Public Health.

[31] Seedat F., Hargreaves S., Nellums L.B., Ouyang J., Brown M., Friedland J.S. (2018). How effective are approaches to migrant screening for infectious diseases in Europe? A systematic review. Lancet Infect. Dis..

[32] Kortas A., Polenz J., von Hayek J., Rüdiger S., Rottbauer W., Storr U., Wibmer T. (2017). Screening for infectious diseases among asylum seekers newly arrived in Germany in 2015: A systematic single-centre analysis. Public Health.

[33] Aldridge R.W., Yates T.A., Zenner D., White P.J., Abubakar I., Hayward A.C. (2014). Pre-entry screening programmes for tuberculosis in migrants to low-incidence countries: A systematic review and meta-analysis. Lancet Infect. Dis..

[34] Carballo M., Hargreaves S., Gudumac I., Maclean E.C. (2017). Evolving migrant crisis in Europe: Implications for health systems. Lancet Glob. Health.

[35] Karki T., Napoli C., Riccardo F., Fabiani M., Dente M.G., Carballo M., Noori T., Declich S. (2014). Screening for infectious diseases among newly arrived migrants in EU/EEA countries-varying practices but consensus on the utility of screening. Int. J. Environ. Res. Public Health.

[36] Semenza J.C., Carrillo-Santisteve P., Zeller H., Sandgren A., van der Werf M.J., Severi E., Pastore Celentano L., Wiltshire E., Suk J.E., Dinca I. (2016). Public Health needs of migrants, refugees and asylum seekers in Europe, 2015: Infectious disease aspects. Eur. J. Public Health.

[37] Schunemann H.J., Wiercioch W., Brozek J., Etxeandia-Ikobaltzeta I., Mustafa R.A., Manja V., Brignardello-Petersen R., Neumann I., Falavigna M., Alhazzani W. (2017). GRADE Evidence to Decision (EtD) frameworks for adoption, adaptation, and de novo development of trustworthy recommendations: GRADE-ADOLOPMENT. J. Clin. Epidemiol..

[38] Moher D., Liberati A., Tetzlaff J., Altman D.G., Group P. (2009). Preferred reporting items for systematic reviews and meta-analyses: The PRISMA statement. PLoS Med..

[39] Pottie K., Mayhew A.D., Morton R.L., Greenaway C., Akl E.A., Rahman P., Zenner D., Pareek M., Tugwell P., Welch V. (2017). Prevention and assessment of infectious diseases among children and adult migrants arriving to the European Union/European Economic Association: A protocol for a suite of systematic reviews for public health and health systems. BMJ Open.

[40] Shemilt I., Thomas J., Morciano M. (2010). A web-based tool for adjusting costs to a specific target currency and price year. Evid. Policy A J. Res. Debate Pract..

[41] Shea B.J., Grimshaw J.M., Wells G.A., Boers M., Andersson N., Hamel C., Porter A.C., Tugwell P., Moher D., Bouter L.M. (2007). Development of AMSTAR: A measurement tool to assess the methodological quality of systematic reviews. BMC Med. Res. Methodol..

[42] The Newcastle-Ottawa Scale (NOS) for Assessing the Quality of Nonrandomised Studies in Meta-Analyses. http://www.ohri.ca/programs/clinical_epidemiology/oxford.asp.

[43] Whiting P.F., Rutjes A.W., Westwood M.E., Mallett S., Deeks J.J., Reitsma J.B., Leeflang M.M., Sterne J.A., Bossuyt P.M. (2011). QUADAS-2: A revised tool for the quality assessment of diagnostic accuracy studies. Ann. Intern. Med..

[44] Ochodo E.A., Gopalakrishna G., Spek B., Reitsma J.B., van Lieshout L., Polman K., Lamberton P., Bossuyt P.M.M., Leeflang M.M.G. (2015). Circulating antigen tests and urine reagent strips for diagnosis of active schistosomiasis in endemic areas. Cochrane Database Syst. Rev..

[45] King C.H., Bertsch D. (2013). Meta-analysis of Urine Heme Dipstick Diagnosis of *Schistosoma haematobium* Infection, Including Low-Prevalence and Previously-Treated Populations. PLoS Negl. Trop. Dis..

[46] Wang W., Li Y., Li H., Xing Y., Qu G., Dai J., Liang Y. (2012). Immunodiagnostic efficacy of detection of Schistosoma japonicum human infections in China: A meta analysis. Asian Pac. J. Trop. Med..

[47] Danso-Appiah A., Olliaro P.L., Donegan S., Sinclair D., Utzinger J. (2013). Drugs for treating Schistosoma mansoni infection. Cochrane Database Syst. Rev..

[48] Kramer C.V., Zhang F., Sinclair D., Olliaro P.L. (2014). Drugs for treating urinary schistosomiasis. Cochrane Database Syst. Rev..

[49] Pérez del Villar L., Burguillo F.J., López-Abán J., Muro A. (2012). Systematic Review and Meta-Analysis of Artemisinin Based Therapies for the Treatment and Prevention of Schistosomiasis. PLoS ONE.

[50] Yang F., Tan X.D., Liu B., Yang C., Ni Z.L., Gao X.D., Wang Y. (2015). Meta-analysis of the diagnostic efficiency of the questionnaires screening for schistosomiasis. Parasitol. Res..

[51] Campo Polanco L., Gutierrez L.A., Cardona Arias J. (2014). Diagnosis of Strongyloides Stercoralis infection: Meta-analysis on evaluation of conventional parasitological methods (1980–2013). Rev. Esp. Salud Publica.

[52] Henriquez-Camacho C., Gotuzzo E., Echevarria J., White A.C., Terashima A., Samalvides F., Pérez-Molina J.A., Plana M.N. (2016). Ivermectin versus albendazole or thiabendazole for Strongyloides stercoralis infection. Cochrane Database Syst. Rev..

[53] Danso-Appiah A., Minton J., Boamah D., Otchere J., Asmah R.H., Rodgers M., Bosompem K.M., Eusebi P., De Vlas S.J. (2016). Accuracy of point-of-care testing for circulatory cathodic antigen in the detection of schistosome infection: Systematic review and meta-analysis. Bull. World Health Organ..

[54] Kinkel H.F., Dittrich S., Baumer B., Weitzel T. (2012). Evaluation of eight serological tests for diagnosis of imported schistosomiasis. Clin. Vaccine Immunol..

[55] Lodh N., Mwansa J.C., Mutengo M.M., Shiff C.J. (2013). Diagnosis of Schistosoma mansoni without the stool: Comparison of three diagnostic tests to detect Schistosoma [corrected] mansoni infection from filtered urine in Zambia. Am. J. Trop. Med. Hyg..

[56] Espirito-Santo M.C., Alvarado-Mora M.V., Dias-Neto E., Botelho-Lima L.S., Moreira J.P., Amorim M., Pinto P.L., Heath A.R., Castilho V.L., Goncalves E.M. (2014). Evaluation of real-time PCR assay to detect Schistosoma mansoni infections in a low endemic setting. BMC Infect. Dis..

[57] Espirito-Santo M.C., Alvarado-Mora M.V., Pinto P.L., Sanchez M.C., Dias-Neto E., Castilho V.L., Goncalves E.M., Chieffi P.P., Luna E.J., Pinho J.R. (2015). Comparative Study of the Accuracy of Different Techniques for the Laboratory Diagnosis of Schistosomiasis Mansoni in Areas of Low Endemicity in Barra Mansa City, Rio de Janeiro State, Brazil. Biomed. Res. Int..

[58] da Frota S.M., Carneiro T.R., Queiroz J.A., Alencar L.M., Heukelbach J., Bezerra F.S. (2011). Combination of Kato-Katz faecal examinations and ELISA to improve accuracy of diagnosis of intestinal schistosomiasis in a low-endemic setting in Brazil. Acta Trop..

[59] Silveira A.M., Costa E.G., Ray D., Suzuki B.M., Hsieh M.H., Fraga L.A., Caffrey C.R. (2016). Evaluation of the CCA Immuno-Chromatographic Test to Diagnose Schistosoma mansoni in Minas Gerais State, Brazil. PLoS Negl. Trop. Dis..

[60] Espirito-Santo M.C., Sanchez M.C., Sanchez A.R., Alvarado-Mora M.V., Castilho V.L., Goncalves E.M., Luna E.J., Gryschek R.C. (2014). Evaluation of the sensitivity of IgG and IgM ELISA in detecting Schistosoma mansoni infections in a low endemicity setting. Eur. J. Clin. Microbiol. Infect. Dis..

[61] Beltrame A., Guerriero M., Angheben A., Gobbi F., Requena-Mendez A., Zammarchi L., Formenti F., Perandin F., Buonfrate D., Bisoffi Z. (2017). Accuracy of parasitological and immunological tests for the screening of human schistosomiasis in immigrants and refugees from African countries: An approach with Latent Class Analysis. PLoS Negl. Trop. Dis..

[62] Bisoffi Z., Buonfrate D., Sequi M., Mejia R., Cimino R.O., Krolewiecki A.J., Albonico M., Gobbo M., Bonafini S., Angheben A. (2014). Diagnostic accuracy of five serologic tests for Strongyloides stercoralis infection. PLoS Negl. Trop. Dis..

[63] Rascoe L.N., Price C., Shin S.H., McAuliffe I., Priest J.W., Handali S. (2015). Development of Ss-NIE-1 recombinant antigen based assays for immunodiagnosis of strongyloidiasis. PLoS Negl. Trop. Dis..

[64] Knopp S., Salim N., Schindler T., Karagiannis Voules D.A., Rothen J., Lweno O., Mohammed A.S., Singo R., Benninghoff M., Nsojo A.A. (2014). Diagnostic accuracy of Kato-Katz, FLOTAC, Baermann, and PCR methods for the detection of light-intensity hookworm and Strongyloides stercoralis infections in Tanzania. Am. J. Trop. Med. Hyg..

[65] King C.H., Olbrych S.K., Soon M., Singer M.E., Carter J., Colley D.G. (2011). Utility of Repeated Praziquantel Dosing in the Treatment of Schistosomiasis in High-Risk Communities in Africa: A Systematic Review. PLoS Negl. Trop. Dis..

[66] Muennig P., Pallin D., Sell R.L., Chan M.-S. (1999). The Cost Effectiveness of Strategies for the Treatment of Intestinal Parasites in Immigrants. N. Engl. J. Med..

[67] Muennig P., Pallin D., Challah C., Khan K. (2004). The cost-effectiveness of ivermectin vs. albendazole in the presumptive treatment of strongyloidiasis in immigrants to the United States. Epidemiol. Infect..

[68] Maskery B., Coleman M.S., Weinberg M., Zhou W., Rotz L., Klosovsky A., Cantey P.T., Fox L.M., Cetron M.S., Stauffer W.M. (2016). Economic Analysis of the Impact of Overseas and Domestic Treatment and Screening Options for Intestinal Helminth Infection among US-Bound Refugees from Asia. PLoS Negl. Trop. Dis..

[69] Worrell C.M., Bartoces M., Karanja D.M., Ochola E.A., Matete D.O., Mwinzi P.N., Montgomery S.P., Secor W.E. (2015). Cost analysis of tests for the detection of Schistosoma mansoni infection in children in western Kenya. Am. J. Trop. Med. Hyg..

[70] Libman M.D., MacLean J.D., Gyorkos T.W. (1993). Screening for schistosomiasis, filariasis, and strongyloidiasis among expatriates returning from the tropics. Clin. Infect. Dis..

[71] CDC (2013). Guidelines for Overseas Presumptive Treatment of Strongyloidiasis, Schistosomiasis, and Soil-Transmitted Helminth Infections.

[72] Buonfrate D., Sequi M., Mejia R., Cimino R.O., Krolewiecki A.J., Albonico M., Degani M., Tais S., Angheben A., Requena-Mendez A. (2015). Accuracy of five serologic tests for the follow up of Strongyloides stercoralis infection. PLoS Negl. Trop. Dis..

[73] Zammarchi L., Bonati M., Strohmeyer M., Albonico M., Requena-Méndez A., Bisoffi Z., Nicoletti A., García H.H., Bartoloni A. (2017). Screening, diagnosis and management of human cysticercosis and T. solium taeniasis: Technical recommendations by the COHEMI project study group. Trop. Med. Int. Health.

[74] Jonas D.E., Ferrari R.M., Wines R.C., Vuong K.T., Cotter A., Harris R.P. (2018). Evaluating evidence on intermediate outcomes: Considerations for groups making healthcare recommendations. Am. J. Prev. Med..

[75] Atkins D., Best D., Briss P.A., Eccles M., Falck-Ytter Y., Flottorp S., Guyatt G.H., Harbour R.T., Haugh M.C., Henry D. (2004). Grading quality of evidence and strength of recommendations. BMJ.

